# Personal light exposure patterns and incidence of type 2 diabetes: analysis of 13 million hours of light sensor data and 670,000 person-years of prospective observation

**DOI:** 10.1016/j.lanepe.2024.100943

**Published:** 2024-06-05

**Authors:** Daniel P. Windred, Angus C. Burns, Martin K. Rutter, Chris Ho Ching Yeung, Jacqueline M. Lane, Qian Xiao, Richa Saxena, Sean W. Cain, Andrew J.K. Phillips

**Affiliations:** a Turner Institute for Brain and Mental Health, School of Psychological Sciences, Faculty of Medicine, Nursing and Health Sciences, Monash University, Melbourne, VIC, Australia; b Division of Sleep and Circadian Disorders, Brigham and Women's Hospital, Boston, MA, USA; c Division of Sleep Medicine, Harvard Medical School, Boston, MA, USA; d Program in Medical and Population Genetics, Broad Institute, Cambridge, MA, USA; e Center for Genomic Medicine, Massachusetts General Hospital, Boston, MA, USA; f Centre for Biological Timing, Division of Endocrinology, Diabetes & Gastroenterology, School of Medical Sciences, Faculty of Biology, Medicine and Health, Manchester Academic Health Science Centre, University of Manchester, Manchester, UK; g Diabetes, Endocrinology and Metabolism Centre, NIHR Manchester Biomedical Research Centre, Manchester University NHS Foundation Trust, Manchester, UK; h Department of Epidemiology, Human Genetics and Environmental Sciences, School of Public Health, The University of Texas Health Science Center at Houston, Houston, TX, United States; i Center for Spatial-temporal Modeling for Applications in Population Sciences, School of Public Health, The University of Texas Health Science Center at Houston, Houston, TX, United States; j Department of Anesthesia, Critical Care and Pain Medicine, Massachusetts General Hospital and Harvard Medical School, Boston, MA, USA; k Flinders Health and Medical Research Institute (Sleep Health), Flinders University, Bedford Park, SA, Australia

**Keywords:** Light sensor, Light at night, Sleep, Circadian, Circadian disruption, Type 2 diabetes, Metabolic disease, Cardiometabolic, Prospective, UK biobank

## Abstract

**Background:**

Light at night disrupts circadian rhythms, and circadian disruption is a risk factor for type 2 diabetes. Whether personal light exposure predicts diabetes risk has not been demonstrated in a large prospective cohort. We therefore assessed whether personal light exposure patterns predicted risk of incident type 2 diabetes in UK Biobank participants, using ∼13 million hours of light sensor data.

**Methods:**

Participants (N = 84,790, age (M ± SD) = 62.3 ± 7.9 years, 58% female) wore light sensors for one week, recording day and night light exposure. Circadian amplitude and phase were modeled from weekly light data. Incident type 2 diabetes was recorded (1997 cases; 7.9 ± 1.2 years follow-up; excluding diabetes cases prior to light-tracking). Risk of incident type 2 diabetes was assessed as a function of day and night light, circadian phase, and circadian amplitude, adjusting for age, sex, ethnicity, socioeconomic and lifestyle factors, and polygenic risk.

**Findings:**

Compared to people with dark nights (0–50th percentiles), diabetes risk was incrementally higher across brighter night light exposure percentiles (50–70th: multivariable-adjusted HR = 1.29 [1.14–1.46]; 70–90th: 1.39 [1.24–1.57]; and 90–100th: 1.53 [1.32–1.77]). Diabetes risk was higher in people with lower modeled circadian amplitude (aHR = 1.07 [1.03–1.10] per SD), and with early or late circadian phase (aHR range: 1.06–1.26). Night light and polygenic risk independently predicted higher diabetes risk. The difference in diabetes risk between people with bright and dark nights was similar to the difference between people with low and moderate genetic risk.

**Interpretation:**

Type 2 diabetes risk was higher in people exposed to brighter night light, and in people exposed to light patterns that may disrupt circadian rhythms. Avoidance of light at night could be a simple and cost-effective recommendation that mitigates risk of diabetes, even in those with high genetic risk.

**Funding:**

Australian Government Research Training Program.

**Research in context**

**Evidence before this study**

We searched PubMed and Google Scholar for studies published up to December 2023, using search terms (“light” OR “light at night” OR “light exposure” OR “circadian”) AND “diabetes”. Small-scale observational studies linked type 2 diabetes and associated pathophysiology with night light exposure recorded by personal and bedroom light sensors. Larger cohort studies linked outdoor night light assessed from satellite data with higher incidence and prevalence of type 2 diabetes, but did not assess personal light exposure. Experimental studies in animals and humans demonstrated that exposure to light patterns that disrupt circadian rhythms caused reduced glucose tolerance, altered insulin secretion and lipid profiles, and weight gain, supporting the role of light exposure in the pathogenesis of type 2 diabetes.

**Added value of this study**

This was the largest known study to link personal light exposure with risk of type 2 diabetes, analyzing ∼13 million hours of light sensor data and incident diabetes across ∼670,000 person-years of follow-up, in ∼85,000 individuals. Light sensors allowed for approximation of personal day and night light exposure, in contrast with the satellite-derived outdoor night light environments analysed in previous cohort studies. The cohort was well-characterized, and consisted of older individuals who were at higher risk of disrupted circadian rhythms and incident type 2 diabetes. Polygenic risk scores supported independent contributions of genetic susceptibility and light exposure to risk of type 2 diabetes. Circadian rhythm modeling supported circadian disruption as a linking mechanism between light exposure and type 2 diabetes, and demonstrated that computational estimations of circadian rhythms have predictive utility for cardiometabolic health.

**Implications of all the available evidence**

Exposure to brighter night light, and light patterns that disrupt circadian rhythms, predict higher risk of type 2 diabetes in older adults. Advising people to avoid night light is a simple and cost-effective recommendation that may ease the global health burden of type 2 diabetes.

## Introduction

Circadian rhythm disruption^1^ has been strongly implicated in the development of type 2 diabetes.^2^ Circadian rhythms are disrupted by light exposure at night, which shifts the timing (phase-shift) and weakens the signal (amplitude suppression) of the central circadian pacemaker in the hypothalamus.^1^^,^^3^^,^^4^ This central pacemaker orchestrates the timing of metabolic processes required for glucose homeostasis,^5^^,^^6^ including circadian rhythms in insulin secretory capacity that peak during the day,^7^^,^^8^ and circadian rhythms in glucose secretion that peak at night.^9^, ^10^, ^11^, ^12^ Mismatch of internal circadian rhythms with external environmental and behavioral rhythms can cause a pre-diabetic state in healthy humans.^12^ Prolonged exposure to internal-external mismatch is associated with higher risk of type 2 diabetes in shift workers^13^^,^^14^ and people with social jetlag.^15^ Since light exposure patterns are a modifiable external factor that affects internal circadian physiology, they may also be a modifiable risk factor for the development of type 2 diabetes.

Emerging research demonstrates that light at night is linked with cardiometabolic pathophysiology, including type 2 diabetes. Higher risks for type 2 diabetes,^16^ obesity, and hypertension^17^ have been observed in people with greater exposure to night light, measured with wrist-worn,^17^ and bedside^16^ light sensors in small cohort studies, and experimental exposure to light during sleep has been shown to increase next-day insulin resistance.^18^ Experimental work in animal models supports night light exposure and circadian disruption as causal factors in diabetes pathophysiology. Reduced glucose tolerance, altered insulin secretion, and weight gain occur in mice exposed to light during the biological night, after controlling for physical activity and food intake,^19^^,^^20^ and circadian clock-mutant mice have altered insulin, glucose, and lipid profiles, and higher obesity, compared with wild type.^21^ Large-scale cohort studies in humans have recently linked night light assessed from satellite data with the incidence^22^ and prevalence^23^ of type 2 diabetes. However, satellite data do not capture the personal indoor lighting environment, where most people spend over 80% of their time. To our knowledge, no large-scale study has examined whether objective, personal light exposure is linked with risk of developing type 2 diabetes, or assessed the role of circadian disruption in this relationship.

We assessed whether risk of incident type 2 diabetes was associated with exposure to light at night, and with modeled circadian amplitude and phase, in 84,790 UK Biobank participants using 13 million hours of data from wrist-worn light sensors, and type 2 diabetes diagnoses from hospital, primary-care, and death register records across 7.9 ± 1.2 years of follow-up.

## Methods

### Overview

The UK Biobank cohort consists of approximately 502,000 participants, aged between 40 and 69 years at time of recruitment (2006–2010).^24^ Participants were invited to participate based on age criteria alone. Assessment locations were chosen to capture the socio-economic, ethnic, and urban-rural composition of the UK population. Several studies have shown that assessments of exposure-disease relationships in the UK Biobank are widely generalizable.^25^ A sub-cohort of 103,669 participants wore Axivity AX3 devices (Axivity, Newcastle upon Tyne, UK) with light sensors on their dominant wrist, for one week between 2013 and 2016. Devices were distributed and returned by post, and participants were instructed to wear devices throughout the entire week (S1). Incident type 2 diabetes diagnoses were recorded between participant light-tracking and study end date of 19th December, 2022. Ethical approval was received by the UK Biobank from the North West Multi-centre Research Ethics Committee (S1).

### Exposure: light and modeled circadian rhythms

Light exposure was captured by wrist-worn Axivity AX3 devices, which contained a silicon photodiode light sensor (APDS9007), with peak sensitivity wavelength of 560 nm. Devices had an approximately linear response to illuminance between 0 and 5500 lx, as reported in our previous work.^26^ Devices logged light and accelerometer data at 100 Hz.

Light data from AX3 devices were subject to several cleaning and processing steps prior to final analyses (see S4). Light data captured during periods of device non-wear were removed. Non-wear of devices was determined using GGIR, a validated R package for determining sleep-wake from accelerometer data,^27^ as previously reported.^28^^,^^29^ After non-wear exclusion, participants had a median (IQR) of 6.90 (5.95–6.96) days of light data remaining. Participants without valid accelerometer data were excluded, as defined by GGIR, due to data corruption, or persistent non-wear (8004 of 103,669).

Night light and day light exposures were derived for each participant from their one-week light recording, by extracting 24-h light profiles, and by applying factor analysis to these profiles. Twenty-four-hour light profiles were extracted by binning weekly data into 48 half-hour clock time intervals for each participant (e.g., all light data collected between 12:00–12:30). As previously reported,^26^ factor analysis was applied to the set of 48 half-hour clock time intervals, assessing the variance structure of light data across the day and night in an unsupervised manner. A two-factor structure of day light (07:30–20:30) and night light (00:30–06:00) was extracted. Night and day light intensity values were derived by taking the mean of recorded light exposure for each participant within these clock time ranges. Participant-level light data cleaning rules were applied, excluding those with low-quality data (6761 of 95,665; see S12).

Circadian phase and amplitude were modeled from light data. For each participant, their one-week light time series was input to an established mathematical model^30^ of the response of human photoreceptors and the central circadian pacemaker to light exposure. This model has been applied in a wide range of populations, including healthy adults, shift workers, and some clinical populations,^31^ and captures the phase-amplitude response of the human central circadian pacemaker to light.^4^ In the model, circadian phase and amplitude change dynamically over time, depending upon the pattern of light exposure. Light exposure close to the model-defined circadian minimum (i.e., the middle of the ‘biological night’) suppresses amplitude, while light exposure early or late in the biological night causes phase delays or phase advances, respectively. Model equations and implementation are described in detail in S12. Phase was calculated for each approximate 24-h period, and represented the predicted time of minimum core body temperature. Mean and standard deviation of phase were calculated for each participant across the week. Mean, minimum, and maximum amplitudes were calculated across the week for each participant.

### Outcome: incident type 2 diabetes

Participants diagnosed with type 2 diabetes prior to light-tracking were excluded, and diagnoses were derived from combined self-report, hospital admissions, primary care, and death register data. Incident diagnoses of type 2 diabetes were derived from hospital admission records, primary care, and death register (see S1) between light-tracking and study end date (19th December, 2022). In non-diagnosed individuals, time-to-event data were right-censored at time of participant mortality or study end date. Records were in accordance with ICD-10 code E11, and were described as ‘non-insulin dependent diabetes’ in the UK Biobank records.

### Covariates

Covariates included sex, age at time of light-tracking, self-reported ethnic background, employment status, yearly household income, Townsend Deprivation Index (average material deprivation associated with a participant's residential location), education level, urbanicity (residential location >10,000 population), smoking status (never/previous/current), alcohol consumption (days per week), healthy diet score (derived according to dietary recommendations for cardiometabolic health^32^), shift work status, chronotype, mental health symptoms, and self-reported engagement with mental health services, and were collected between 2006 and 2010. Physical activity was included as the average acceleration across each weekly recording, and was extracted in previous work.^33^ Physical measurements included body mass index (BMI), systolic and diastolic blood pressure, and assays for high-density lipoprotein, low-density lipoprotein, triglyceride, glucose, and HbA1c levels, collected between 2006 and 2010. Sleep duration was estimated using a validated R package for estimating sleep-wake state from accelerometer data, as reported previously.^27^^,^^29^ Photoperiod was derived as the interval between sunrise and sunset on the date of light-tracking at 53.4808° N, 2.2426° W (Manchester, UK). Covariates are described in detail in S2 and S3, and missing data are reported in S5.

### Polygenic risk score of type 2 diabetes

A polygenic risk score (PRS) for type 2 diabetes^34^ was constructed using PRS-continuous shrinkage (PRS-CS).^35^ PRS–CS applies a continuous shrinkage prior on SNP effect sizes within a Bayesian framework and models linkage disequilibrium to improve polygenic prediction. The type 2 diabetes PRS was then scored in UK Biobank participants using PLINK 2^36^ as the weighted sum of the effect alleles, using the following formula:

$$S_{i\;}=\sum_{j=1}^{M}{\hat{\beta }}_{j}g_{ij}$$

where $S_{i\;}$ is the polygenic score for individual *i*, ${\hat{\beta }}_{j}$ is the weighted additive effect of the effect allele at SNP *j*, and $g_{ij}$ is the genotype for individual *i* at SNP *j*.

### Statistical analysis

Hazard ratios for incident type 2 diabetes were estimated using Cox proportional hazards models, with night light and day light included as additive predictors, and time since light-tracking used as the timescale. Night light and day light data were both grouped into four percentile ranges: 0–50% (referent group), 50–70%, 70–90%, and 90–100%. For night light, the 0–50th percentile group contained individuals in dark environments across the night (<1 lx), and this group was hypothesized to have the lowest risk of type 2 diabetes. Bins were of unequal size due to the skewed distribution of the light data. Data included in each percentile group were greater than or equal to the lower bound and less than the upper bound of their respective percentile ranges.

Modeled circadian variables were included as predictors of diabetes risk in individual Cox models. Mean, minimum, and maximum circadian amplitude, and phase variability, were z-scored and included as linear predictors of diabetes risk. Mean circadian phase was split into quintiles, and included as a categorical predictor of diabetes risk: the 40–60th percentile group was centered at the sample circular mean phase (03:50 h) and used as the referent group, accounting for the circular nature of phase data.

Polygenic risk scores for type 2 diabetes were divided into quartiles, and included in Cox models with and without light exposure, to assess the independence of light exposure and genetic risk as predictors of incident type 2 diabetes. For all genetic analyses we restricted the sample to individuals of European ancestry and additionally adjusted for the top 5 principal components of ancestry, to control for potential residual population stratification within the European ancestry sub-population (see S12 for additional detail).

Models predicting incident type 2 diabetes risk from night light and day light, modeled circadian variables, and polygenic risk, were subject to three levels of adjustment for potentially confounding factors: Model 1 was adjusted for age, sex, and ethnicity; Model 2 was additionally adjusted for income, material deprivation, education, and employment status; and Model 3 was further adjusted for smoking status, alcohol consumption, healthy diet, physical activity, and urbanicity. The proportional hazards assumption was assessed across these models (S11). Further detail on model implementation is included in S12.

### Supplementary analysis

Models predicting incident type 2 diabetes risk from night light and day light were subject to several additional adjustments. Model 3 was additionally adjusted for cardiometabolic risk factors (high BMI, hypertension, and high cholesterol ratio), sleep duration, chronotype, photoperiod at light-tracking, and mental health (depression and anxiety symptoms, and self-reported engagement with mental health services) (S6). Model 3 was tested in a sub-sample of participants who were not shift workers (S6). Models 1–3 were tested after exclusion of participants with pre-diabetes prior to light-tracking, defined as HbA1c ≥ 39 mmol/mol or random blood glucose ≥7.8 mmol/L (S8). The interaction between participant sex and night light exposure was tested across Models 1–3, to assess whether relationships between light exposure and type 2 diabetes differed between males and females (S7). Proportional sub-hazards of incident type 2 diabetes were assessed across Models 1–3, incorporating participant mortality as a competing risk for developing diabetes (S9).^37^ Finally, type 2 diabetes risk was predicted from light exposures across 24 h, for Models 1–3 (S10).

### Role of the funding source

The funding source played no role in design, analysis, interpretation, or report writing for this study.

## Results

### Descriptive statistics

Analyses were completed for 84,790 participants (see Table 1) with complete daily light profiles, and without type 2 diabetes prior to light-tracking (4085 cases excluded). Light profiles in final analyses were derived from a mean (±SD) of 6.92 ± 0.54 days of complete light data per participant. Mean (±SD) follow up period was 7.91 ± 1.2 years from light-tracking to study endpoint, and the maximum follow-up period was 9.55 years. There were 1997 observed cases of type 2 diabetes across 670,844 person-years of follow-up (2.98 cases per 1000 person-years). Number and percentage of diabetes cases for light exposure percentile groups are detailed in Table 2. A distribution of light exposures across 24 h is provided in our previous work.^26^

**Table 1:** Participant characteristics by level of night and day light exposure.

	Night light exposure percentile				Day light exposure percentile			
	0–50%	50–70%	70–90%	90–100%	0–50%	50–70%	70–90%	90–100%
Age								
M ± SD	62.6 ± 7.86	61.7 ± 7.91	61.9 ± 7.79	62.2 ± 7.69	61.9 ± 7.95	62.3 ± 7.84	62.5 ± 7.74	63.5 ± 7.43
Range	43.5–78.9	43.5–79.0	43.7–78.0	43.8–78.4	43.6–79.0	43.6–78.7	43.5–78.8	43.5–78.5
Sex, % male (N)	40.5 (17,302)	44.2 (7495)	43.8 (7358)	44.2 (3691)	41.5 (17,544)	41.0 (6946)	42.5 (7228)	48.3 (4128)
Ethnicity, % white (N)	97.8 (41,637)	96.9 (16,365)	96.4 (16,141)	95.7 (7951)	96.5 (40,704)	97.2 (16,416)	97.8 (16,563)	98.8 (8411)
Employment status, % employed (N)	60.2 (25,545)	65.1 (10,973)	65.9 (10,991)	64.8 (5360)	64.7 (27,179)	62.4 (10,501)	61.2 (10,328)	57.3 (4861)
Income								
% <£18 k (N)	12.5 (5298)	12.8 (2148)	12.5 (2086)	13.6 (1124)	13.1 (5488)	12.6 (2111)	12.3 (2075)	11.5 (982)
% £18–29.9 k (N)	22.0 (9304)	21.2 (3569)	20.9 (3493)	21.7 (1802)	21.4 (9011)	21.9 (3675)	21.4 (3616)	21.9 (1866)
% £30–51.9 k (N)	26.1 (11,065)	26.3 (4434)	26.1 (4366)	25.2 (2092)	26.1 (10,958)	25.6 (4297)	26.4 (4458)	26.4 (2244)
% £52-100 k (N)	22.9 (9725)	23.4 (3934)	23.7 (3956)	22.6 (1876)	23.1 (9716)	23.0 (3875)	23.3 (3938)	23.1 (1962)
% >£100 k (N)	6.40 (2712)	7.10 (1196)	7.36 (1229)	7.52 (623)	6.79 (2854)	6.66 (1120)	7.04 (1189)	7.02 (597)
Education								
% other (non-university) (N)	47.9 (20,221)	48.3 (8109)	48.4 (8057)	48.8 (4032)	47.6 (19,922)	48.5 (8129)	48.5 (8162)	49.7 (4206)
% university/college (N)	43.9 (18,545)	43.9 (7369)	44.0 (7319)	43.3 (3577)	44.5 (18,660)	43.7 (7317)	43.4 (7306)	41.6 (3527)
Townsend Deprivation Index								
M ± SD	−1.94 ± 2.69	−1.71 ± 2.81	−1.64 ± 2.86	−1.41 ± 2.97	−1.61 ± 2.88	−1.81 ± 2.75	−1.93 ± 2.69	−2.27 ± 2.43
Range	−6.26 to 10.5	−6.26 to 9.89	−6.26 to 9.99	−6.26 to 9.89	−6.26 to 10.5	−6.26 to 9.89	−6.26 to 9.89	−6.26 to 8.94
Smoking								
% previous (N)	34.2 (14,564)	35.6 (6023)	37.4 (6270)	38.4 (3196)	34.6 (14,594)	35.6 (6010)	36.4 (6173)	38.4 (3276)
% current (N)	5.40 (2298)	7.17 (1212)	8.09 (1357)	10.1 (842)	6.99 (2949)	6.92 (1169)	6.51 (1103)	5.73 (488)
Alcohol (M ± SD, days per week)	3.00 ± 2.48	3.01 ± 2.49	3.02 ± 2.50	2.98 ± 2.56	2.90 ± 2.48	3.01 ± 2.50	3.09 ± 2.51	3.32 ± 2.53
Urbanicity, % >10,000 population (N)	83.1 (35,112)	83.5 (14,036)	85.5 (14,240)	86.3 (7112)	85.3 (35,750)	83.9 (14075)	83.0 (13954)	79.2 (6721)
Physical activity (milli-g)								
M ± SD	28.1 ± 7.86	28.8 ± 8.23	28.7 ± 8.23	28.1 ± 8.24	27.5 ± 7.88	28.4 ± 7.95	29.3 ± 8.04	30.7 ± 8.46
Range	4.83–69.2	7.26–69.3	5.88–69.2	5.50–69.4	5.50–69.3	4.94–67.9	7.55–69.4	4.83–67.4
Diet score, % healthy (N)	26.4 (10,942)	25.1 (4147)	24.7 (4026)	24.5 (1984)	25.1 (10,334)	25.7 (4229)	26.0 (4297)	26.8 (2239)

Light exposure percentile ranges were derived from estimated average illuminance (lux), with 90–100th percentile group representing the group with brightest light exposure. Education categories were ‘none’, ‘other (non-university)’, and ‘university’. Positive and negative TDI values represent locations with relatively higher or lower material deprivation, respectively, compared to areas with average material deprivation (score of zero). Smoking categories were ‘never’, ‘previous’, or ‘current’. Diet score categories were ‘healthy’ and ‘unhealthy’, defined according to meeting ≥5 of 10 dietary intake criteria for cardiometabolic health (see S3).

**Table 2:** Risk of incident type 2 diabetes, by level of night light and day light exposure, including the proportion and number of diabetes cases within each light exposure group.

	Percentile	Cases % (N)	HR [95% CI]	p-value
Model 1 (N = 84,510)				
Night light	0–50% (ref.)	1.98 (835)	–	–
	50–70%	2.52 (426)	1.33 [1.18–1.49]^a^	<0.0001
	70–90%	2.72 (459)	1.44 [1.29–1.62]^a^	<0.0001
	90–100%	3.16 (267)	1.67 [1.45–1.92]^a^	<0.0001
Day light	0–50% (ref.)	2.41 (1017)	–	–
	50–70%	2.43 (410)	0.97 [0.86–1.09]	0.60
	70–90%	2.22 (375)	0.84 [0.75–0.95]^a^	0.0055
	90–100%	2.19 (185)	0.73 [0.62–0.85]^a^	<0.0001
Model 2 (N = 83,052)				
Night light	0–50% (ref.)	1.97 (818)	–	–
	50–70%	2.47 (411)	1.28 [1.14–1.44]^a^	<0.0001
	70–90%	2.70 (449)	1.42 [1.26–1.59]^a^	<0.0001
	90–100%	3.14 (261)	1.60 [1.39–1.84]^a^	<0.0001
Day light	0–50% (ref.)	2.38 (987)	–	–
	50–70%	2.41 (400)	0.99 [0.88–1.11]	0.83
	70–90%	2.23 (371)	0.88 [0.78–0.99]^a^	0.037
	90–100%	2.18 (181)	0.77 [0.65–0.90]^a^	0.0014
Model 3 (N = 80,181)				
Night light	0–50% (ref.)	1.96 (786)	–	–
	50–70%	2.46 (394)	1.29 [1.14–1.46]^a^	<0.0001
	70–90%	2.69 (431)	1.39 [1.24–1.57]^a^	<0.0001
	90–100%	3.16 (253)	1.53 [1.32–1.77]^a^	<0.0001
Day light	0–50% (ref.)	2.35 (942)	–	–
	50–70%	2.39 (384)	1.06 [0.94–1.20]	0.32
	70–90%	2.26 (362)	1.02 [0.90–1.15]	0.78
	90–100%	2.20 (176)	0.98 [0.83–1.16]	0.81

Data are hazard ratios (95% CI). Model covariates: model 1: age, sex, and ethnicity; model 2: model 1 covariates plus income, material deprivation, education, and employment status; and model 3: model 2 covariates plus smoking status, alcohol consumption, healthy diet, physical activity, and urbanicity.

a p < 0.05.

Participant characteristics (see Table 1) were as follows: aged 62.3 ± 7.85 years, 57.7% female, 97.1% white ethnicity, 62.8% employed, median income range of £31,000–51,999, 43.8% with university education, 48.1% with other non-university education, Townsend deprivation score of M(±SD) = −1.78 ± 2.78, 6.75% current smokers, 35.5% previous smokers, alcohol consumption on 3.00±-2.50 days per week, 84.0% from an urban postcode, 25.6% with a ‘healthy’ diet score, and average weekly physical activity of 28.40 ± 8.05 milli-g.

### Brighter light at night predicted higher risk of incident type 2 diabetes

**Fig. 1:**
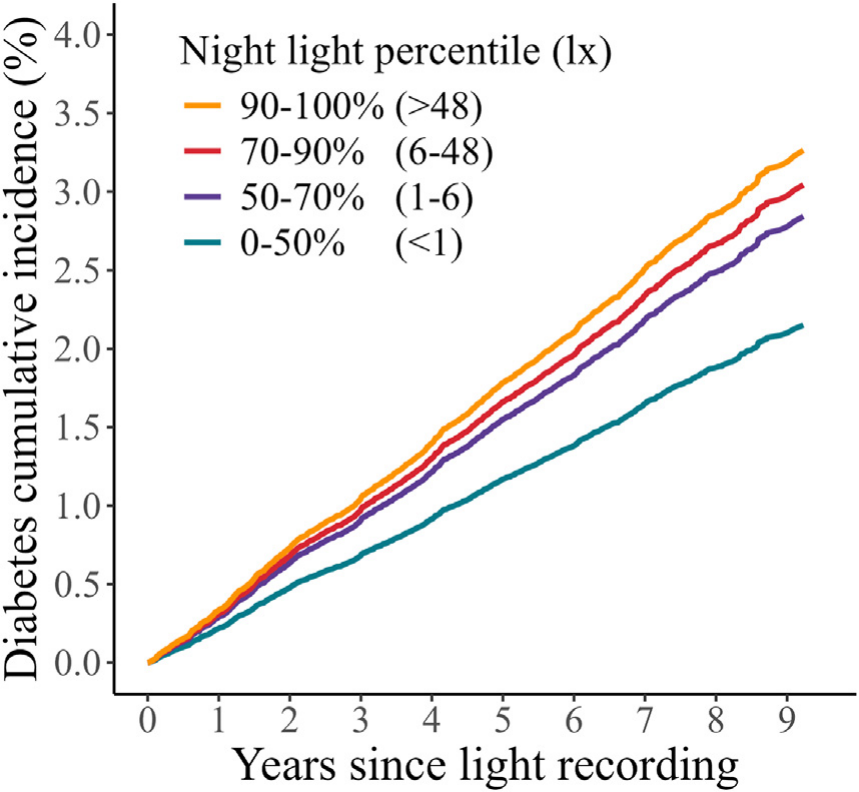
Cumulative incidence of type 2 diabetes by degree of night-light exposure; 0–50%, 50–70%, 70–90%, and 90–100% groups, adjusted for Model 3 covariates.

A dose–response relationship between brighter light at night (00:30–06:00) and higher risk of type 2 diabetes was observed across all levels of model adjustment (Table 2; Fig. 1). Risk of type 2 diabetes was higher in participants with night light exposure between the 90 and 100th percentiles (Model 1: HR = 1.67 [1.45–1.92], p < 0.0001; Model 2: HR = 1.60 [1.39–1.84], p < 0.0001; Model 3: HR = 1.53 [1.32–1.77], p < 0.0001), 70–90th percentiles (Model 1: HR = 1.44 [1.29–1.62], p < 0.0001; Model 2: HR = 1.42 [1.26–1.59], p < 0.0001; Model 3: HR = 1.39 [1.24–1.57], p < 0.0001), and 50–70th percentiles (Model 1: HR = 1.33 [1.18–1.49], p < 0.0001; Model 2: HR = 1.28 [1.14–1.44], p < 0.0001; Model 3: HR = 1.29 [1.14–1.46], p < 0.0001), compared with the darkest 0–50th percentiles.

Light exposure during the day (07:30–20:30) was associated with lower risk of type 2 diabetes in Models 1–2, for participants with exposure between the 90 and 100th percentiles (Model 1: HR = 0.73 [0.62–0.85], p < 0.0001; Model 2: HR = 0.77 [0.65–0.90], p = 0.0014), and between the 70–90th percentiles (Model 1: HR = 0.84 [0.75–0.95], p = 0.0055; Model 2: HR = 0.88 [0.78–0.99], p = 0.037), compared with the darkest 0–50th percentiles. Day light did not significantly predict diabetes risk after additional adjustment for lifestyle factors (Model 3).

Light at night remained a significant predictor of diabetes risk, across the 50–70th, 70–90th, and 90–100th percentiles, after additional adjustments of Model 3 for baseline cardiometabolic risk factors (high BMI, hypertension, and high cholesterol ratio), sleep duration, chronotype, photoperiod, mental health (depression and anxiety symptoms, and self-reported engagement with mental health services), and after exclusion of shift workers (S6). Light at night remained a robust predictor of type 2 diabetes after exclusion of individuals with pre-diabetes prior to light-tracking (S8; aHR range: 1.25–1.49), and in models incorporating competing-risk of mortality (S9; aHR range: 1.29–1.66). Light at night was a robust predictor of type 2 diabetes for both males (aHR range: 1.30–1.57), and females (aHR range: 1.25–1.81), and there were no significant sex-specific differences in light-diabetes relationships (S7).

### Modeled circadian disruption predicted higher risk of incident type 2 diabetes

Higher minimum circadian amplitude robustly predicted a lower type 2 diabetes risk across three levels of model adjustment (Model 1: HR = 0.92 [0.89–0.96], p < 0.0001; Model 2: HR = 0.92 [0.89–0.96], p < 0.0001; Model 3: HR = 0.93 [0.90–0.97], p = 0.00061, per SD, Table 3). Higher mean circadian amplitude predicted lower type 2 diabetes risk across Models 1–2 (Model 1: HR = 0.95 [0.91–1.00], p = 0.031; Model 2: HR = 0.96 [0.92–1.00], p = 0.044, per SD), but not after adjustment for lifestyle factors in Model 3. Intra-individual variability in modeled circadian phase predicted higher risk of diabetes across Models 1–3 (Model 1: HR = 1.04 [1.02–1.07], p = 0.0016; Model 2: HR = 1.04 [1.01-1.07], p = 0.014; Model 3: HR = 1.03 [1.01–1.06], p = 0.017 per SD). Early and late circadian phase quintiles predicted higher risk of type 2 diabetes across three levels of model adjustment compared to the quintile centered at the mean circadian phase of the sample (see Table 4).

**Table 3:** Multivariable-adjusted risks for incident type 2 diabetes associated with modeled circadian rhythm variables.

	Model 1		Model 2		Model 3	
	HR [95% CI]	p-value	HR [95% CI]	p-value	HR [95% CI]	p-value
Mean amplitude	0.95 [0.91–1.00]^a^	0.031	0.96 [0.92–1.00]^a^	0.044	0.99 [0.95–1.04]	0.66
Min. amplitude	0.92 [0.89–0.96]^a^	<0.0001	0.92 [0.89–0.96]^a^	<0.0001	0.93 [0.90–0.97]^a^	0.00061
Max. amplitude	0.99 [0.94–1.03]	0.53	1.00 [0.95–1.04]	0.96	1.05 [1.00–1.10]^a^	0.039
Phase variability	1.04 [1.02–1.07]^a^	0.0016	1.04 [1.01-1.06]^a^	0.011	1.03 [1.01-1.06]^a^	0.017
Mean phase (percentile group)						
0–20%	1.39 [1.20–1.60]^a^	<0.0001	1.25 [1.08–1.45]^a^	0.0034	1.18 [1.01-1.37]^a^	0.032
20–40%	1.30 [1.12–1.50]^a^	0.00059	1.27 [1.09–1.47]^a^	0.0021	1.26 [1.08–1.47]^a^	0.0032
40–60% (ref.)	–	–	–	–	–	–
60–80%	1.07 [0.91–1.25]	0.41	1.10 [0.94–1.29]	0.22	1.06 [0.90–1.24]	0.49
80–100%	1.29 [1.11–1.49]^a^	0.00073	1.30 [1.12–1.50]^a^	0.00069	1.17 [1.01-1.36]^a^	0.042

Data are hazard ratios (95% CIs) and represent differences in risk of incident diabetes associated with a one standard deviation increase in each circadian rhythm variable: mean, min., and max. amplitude, and phase variability. For mean phase, hazard ratios represent the diabetes risk associated with each percentile group relative to the 40–60% reference group (−0.40 to 0.40 h relative to the sample circular mean phase), centered at the sample circular mean phase (03:50 h). Hourly mean phase ranges for the other percentile groups were: −12 to −1.16 h (0–20%), −1.16 to −0.40 h (20–40%), 0.40 to 1.16 h (60–80%), and 1.16 to 12 h (80–100%). Model covariates: model 1: age, sex, and ethnicity; model 2: model 1 covariates plus income, material deprivation, education, and employment status; and model 3: model 2 covariates plus smoking status, alcohol consumption, healthy diet, physical activity, and urbanicity.

a p < 0.05.

**Table 4:** Risk of incident type 2 diabetes predicted by polygenic risk score only, and by polygenic risk score plus light exposure.

	Percentile	PRS only		PRS + light	
		HR [95% CI]	p-value	HR [95% CI]	p-value
Model 1					
Polygenic risk	0–25% (ref.)	–	–	–	–
	25–50%	1.69 [1.44–1.98]^a^	<0.0001	1.69 [1.44–1.98]^a^	<0.0001
	50–75%	2.55 [2.20–2.97]^a^	<0.0001	2.54 [2.18–2.95]^a^	<0.0001
	75–100%	4.68 [4.03–5.43]^a^	<0.0001	4.66 [4.02–5.40]^a^	<0.0001
Night light	0–50% (ref.)	–	–	–	–
	50–70%	–	–	1.35 [1.20–1.53]^a^	<0.0001
	70–90%	–	–	1.41 [1.25–1.60]^a^	<0.0001
	90–100%	–	–	1.73 [1.50–2.00]^a^	<0.0001
Day light	0–50% (ref.)	–	–	–	–
	50–70%	–	–	0.98 [0.87–1.11]	0.74
	70–90%	–	–	0.88 [0.78–1.00]^a^	0.048
	90–100%	–	–	0.74 [0.63–0.87]^a^	0.00033
Model 2					
Polygenic risk	0–25% (ref.)	–	–	–	–
	25–50%	1.66 [1.41–1.95]^a^	<0.0001	1.65 [1.41–1.94]^a^	<0.0001
	50–75%	2.48 [2.13–2.89]^a^	<0.0001	2.46 [2.12–2.87]^a^	<0.0001
	75–100%	4.42 [3.80–5.13]^a^	<0.0001	4.40 [3.78–5.11]^a^	<0.0001
Night light	0–50% (ref.)	–	–	–	–
	50–70%	–	–	1.32 [1.16–1.49]^a^	<0.0001
	70–90%	–	–	1.40 [1.24–1.58]^a^	<0.0001
	90–100%	–	–	1.66 [1.43–1.92]^a^	<0.0001
Day light	0–50% (ref.)	–	–	–	–
	50–70%	–	–	1.00 [0.89–1.13]	0.97
	70–90%	–	–	0.92 [0.82–1.05]	0.21
	90–100%	–	–	0.78 [0.66–0.92]^a^	0.0032
Model 3					
Polygenic risk	0–25% (ref.)	–	–	–	–
	25–50%	1.59 [1.35–1.88]^a^	<0.0001	1.59 [1.35–1.87]^a^	<0.0001
	50–75%	2.36 [2.02-2.75]^a^	<0.0001	2.34 [2.01–2.73]^a^	<0.0001
	75–100%	4.17 [3.58–4.85]^a^	<0.0001	4.14 [3.55–4.82]^a^	<0.0001
Night light	0–50% (ref.)	–	–	–	–
	50–70%	–	–	1.31 [1.16–1.49]^a^	<0.0001
	70–90%	–	–	1.39 [1.23–1.58]^a^	<0.0001
	90–100%	–	–	1.58 [1.36–1.84]^a^	<0.0001
Day light	0–50% (ref.)	–	–	–	–
	50–70%	–	–	1.08 [0.95–1.22]	0.25
	70–90%	–	–	1.07 [0.94–1.21]	0.31
	90–100%	–	–	0.98 [0.83–1.16]	0.82

Data are hazard ratios (95% CI). Exposures are percentiles of type 2 diabetes polygenic risk, night light and day light exposure. Model covariates: model 1: age, sex, ethnicity, and the top five principal components of genetic ancestry; model 2: model 1 covariates plus income, material deprivation, education, and employment status; and model 3: model 2 covariates plus smoking status, alcohol consumption, healthy diet, physical activity, and urbanicity. Models were analysed within a sub-sample of participants with European ancestry only, consisting of 94.6% of participants with complete light data and no type 2 diabetes diagnosis prior to light-tracking.

a p < 0.05.

### Light at night and genetic risk independently predicted incidence of type 2 diabetes

Polygenic risk of type 2 diabetes was a robust predictor of incident type 2 diabetes across Models 1–3 (Table 4). Polygenic risk and night light were robust, independent predictors of incident type 2 diabetes when included together in Models 1–3 as additive predictors (Table 4). The higher diabetes risk observed between adjacent polygenic risk quartiles was comparable in magnitude to the higher diabetes risk observed between bright nights (90–100th percentiles) and dark nights (0–50th percentiles).

## Discussion

Across ∼670,000 person-years of observation in ∼85,000 participants, and 13 million hours of personal light sensor data, we found that exposure to brighter light at night predicted higher risk of incident type 2 diabetes across an average of 7.9 ± 1.2 years of follow-up. Modeling indicated that suppressed circadian amplitude and circadian phase that deviated from the group average also predicted higher risk of type 2 diabetes, supporting the role of circadian disruption in the development of type 2 diabetes. Light exposure at night and polygenic risk were independent predictors of incident type 2 diabetes, indicating that reducing night light may attenuate an individual's risk of type 2 diabetes despite their genetic susceptibility.

We observed a dose-dependent relationship between brighter light exposure at night (00:30 to 06:00) and higher risk of subsequent type 2 diabetes. Compared to individuals with dark environments (the 0–50th percentile), those in the 50–70th, 70–90th, and 90–100th percentiles of light exposure had, respectively, 28–33%, 39–44%, and 53–67% higher risks for developing type 2 diabetes. This relationship between night light and type 2 diabetes was robust to: (i) adjustments for age, sex, ethnicity, socioeconomic status, smoking, alcohol, diet, physical activity, urbanicity, and day light exposure; (ii) additional adjustments for baseline cardiometabolic health, mental health, sleep duration, chronotype, and photoperiod; and (iii) exclusion of shift workers and individuals with pre-diabetic HbA1c or random glucose levels prior to light-tracking. Night light was also a robust predictor of type 2 diabetes within both male and female sub-groups, with no significant difference in this relationship between groups. These findings build on data from longitudinal research that demonstrates higher risk of type 2 diabetes in people exposed to night light, recorded by light sensors.^16^ We confirm these findings in a much larger cohort after controlling for potential confounding factors, and using personal light sensors that capture more than the bedroom environment at night.^16^ Our findings are also consistent with studies of satellite-derived night light in larger cohorts.^22^^,^^23^ These studies demonstrate significant but comparatively weaker relationships between night light and type 2 diabetes (e.g., 7% greater diabetes risk per quintile of brighter night light^23^), possibly reflecting the fact that satellite data do not capture personal light exposure across 24 h.^38^

We applied a validated computational model representing the response of the human central circadian clock to light, to identify participants with weekly light patterns that could suppress the amplitude or shift the phase of their central circadian clock.^3^^,^^4^^,^^39^ Risk of incident diabetes was higher in people with light patterns that could suppress circadian amplitude (7% higher risk per standard deviation reduction in amplitude), and in people with light patterns that could advance circadian phase (18–39%) or delay phase (6–30%) compared to the group average. These results are in keeping with experimental and epidemiological work demonstrating that disrupted circadian rhythms, or exposure to zeitgebers capable of disrupting rhythms, are linked to type 2 diabetes and its associated pathophysiology.^2^^,^^5^^,^^12^^,^^14^^,^^15^ Exposure to light that suppresses or shifts central circadian rhythms to an abnormal phase may alter circadian rhythms in insulin secretory capacity and glucose secretion, by either suppressing these rhythms, or shifting their timing relative to behavioral rhythms in nutritional intake, sleep, and physical activity. For example, disrupted circadian melatonin or glucocorticoid rhythms^40^ may exhibit elevated concentrations during waking hours, thereby reducing pancreatic insulin secretion^41^^,^^42^ and promoting hepatic glucose production^41^ at times that coincide with food intake. Persistent circadian misalignment may lead to persistently elevated postprandial glucose levels, initiating the development of type 2 diabetes by increasing the size and inflammation^43^ of adipocytes, thereby promoting insulin resistance^44^^,^^45^ and the secretion of inflammatory markers (e.g., interleukin-1β) that inhibit pancreatic beta-cell function.^46^

Sleep likely plays an important role in the relationships between light exposure, circadian disruption, and diabetes risk. Sleep and light exposure patterns share a bidirectional relationship, and sleep disruption is an established risk factor for type 2 diabetes.^47^ The relationship between light exposure and diabetes could therefore be partially explained by sleep disruption that co-occurs with night light exposure. Light exposure during the night could lead to disrupted sleep, but awakenings during the night could also lead to greater night light exposure, due to light usage during awakenings. Notably, in our analyses, night light exposure was an independent predictor of type 2 diabetes risk after adjustment for sleep duration. This finding supports night light as a predictor of diabetes risk, independent of sleep duration.

Night light exposure and genetic risk were found to be independent risk factors for developing type 2 diabetes. We derived polygenic risk scores for type 2 diabetes, and confirmed that they were robust predictors of type 2 diabetes diagnoses in the UK Biobank cohort. Higher polygenic scores were associated with 1.6, 2.3, and 4.2 times greater risk of incident diabetes in the second, third, and fourth polygenic risk quartiles, respectively, compared with the lowest-risk quartile. The difference between the 0–50% and 90–100% night light groups was similar to the difference between the 0–25% and 25–50% or the 25–50% and 50–75% polygenic risk categories. This indicates that, while polygenic risk score is a stronger predictor than night light exposure, reducing light exposure at night could attenuate an individual's susceptibility due to genetic risk of developing diabetes. A robust dose-dependent relationship between brighter light at night and higher diabetes risk was observed after adjustment for polygenic risk. This finding indicates that reduction of night light is a potential beneficial strategy for all individuals, including those with high genetic risk.

This study has several limitations. First, we could not investigate the role of food timing, since temporal dietary information was not available. Food timing can alter peripheral circadian rhythms in humans,^48^ impacting glucose tolerance and adiposity,^49^ and may therefore play a key role in the relationships between light, circadian disruption, and diabetes. Second, the cohort studied here had a mean (±SD) age of 62.3 ± 7.85 years, and it is therefore unclear whether our findings generalize to younger cohorts. Third, the computational model of the human circadian pacemaker was originally developed and has primarily been tested in young adults.^4^^,^^50^ Estimated circadian phase and amplitude may therefore not reflect changes in the central circadian clock with age.^51^ Fourth, inter-individual differences in light sensitivity could not be captured. Light intensity required to suppress 50% of melatonin secretion can range from 6 to 350 lux across individuals.^52^ These inter-individual differences in sensitivity of the circadian system to light may contribute to higher variability in the estimated effects of light exposure on type 2 diabetes. Fifth, some socioeconomic factors were captured at the area-level, but not individual level, possibly leading to unmeasured confounding. For example, area-level information on participant housing was captured under urbanicity and deprivation factors, but individual-level housing information was not. An individual's control over their home environment, including the lighting, is a plausible predictor of both night light exposure and type 2 diabetes risk. Sixth, only one week of light data were collected for each individual, and wrist-worn light sensors may have been prone to coverage by individuals' clothing. However, despite these limitations, brighter night light remained a robust predictor of type 2 diabetes even after comprehensive model adjustments. We also note that light exposure patterns were stable in a sub-sample of 2988 participants with repeat-measures, as reported in our previous work.^26^ Seventh, covariates were collected several years prior to light recordings, and some of these covariates may change over time. Finally, relationships between light exposure patterns and diabetes risk in Models 2–3 may be attenuated by mediating pathways. Model 1 may therefore provide a closer approximation of the casual relationship between light exposure patterns and type 2 diabetes risk; however, large-scale intervention studies are required to establish the true causal relationship.

Current behavioral strategies for prevention and treatment of type 2 diabetes focus on increasing physical activity and improving diet, to reduce visceral adiposity and improve diabetes biomarkers.^53^ Our findings show that maintaining a dark environment during the night may mitigate risk of developing type 2 diabetes, likely due to the disruptive effects of light at night on circadian rhythms. Advising people to turn off their lights at night, or use lights that reduce the circadian impact (dim and “warm” light), is a simple, cost-effective, and easily-implementable recommendation that may promote cardiometabolic health and ease the growing global health burden of type 2 diabetes.^54^

## Contributors

DPW, ACB, CHCY, QX, MKR, SWC, and AJKP conceptualized the study. DPW and ACB performed data curation and validation, and conducted formal analyses. MKR, JML, RS, AJKP, and SWC contributed to supervision, project resources, and project administration. DPW, AJKP, and SWC wrote the first draft. All authors reviewed and edited the final manuscript. DPW and ACB have accessed and verified the underlying data reported in this manuscript.

## Data sharing statement

The data used in this manuscript are available in the UK Biobank repository and can be accessed subject to approval by UK Biobank Access Management Team.

## Declaration of interests

AJKP and SWC have received research funding from Delos and Versalux. AJKP and SWC are co-directors of Circadian Health Innovations Pty Ltd. SWC has consulted for Dyson. SWC received research funding from Beacon Lighting. MKR has received consulting fees from Eli Lilly. DPW, ACB, CHCY, JML, QX, and RS declare no competing interests relevant to this manuscript.
