# Supplementary material for: Personal light exposure patterns and incidence of type 2 diabetes: analysis of 13 million hours of light sensor data and 670,000 person-years of prospective observation
Source: Lancet Reg Health Eur. 2024 Jun 5;42:100943. doi: 10.1016/j.lanepe.2024.100943 (PMC11281921; doi:10.1016/j.lanepe.2024.100943)
Supplement: Supplementary Methods and Tables [file mmc1.docx]

**Supplementary Information: Light exposure patterns and incident type 2 diabetes: 13 million hours of light sensor data and 670,000 person-years of prospective observation**

Daniel P. Windred^1^, Angus C. Burns^1-5^, Martin K. Rutter^6,7^, Chris Ho Ching Yeung^8^, Jacqueline M. Lane^2-5^, Qian Xiao^8-9^, Richa Saxena^2-5^, Sean W. Cain ^1^*, & Andrew J. K. Phillips^1^*

**Affiliation / Institution:**

^1^Turner Institute for Brain and Mental Health, School of Psychological Sciences, Faculty of Medicine, Nursing and Health Sciences, Monash University, Melbourne, VIC, Australia

^2^Division of Sleep and Circadian Disorders, Brigham and Women’s Hospital, Boston, MA, USA

^3^Program in Medical and Population Genetics, Broad Institute, Cambridge, MA, USA

^4^Center for Genomic Medicine, Massachusetts General Hospital, Boston, MA, USA

^5^Department of Anesthesia, Critical Care and Pain Medicine, Massachusetts General Hospital and Harvard Medical School, Boston, MA, USA

^6^Centre for Biological Timing, Division of Endocrinology, Diabetes & Gastroenterology, School of Medical Sciences, Faculty of Biology, Medicine and Health, Manchester Academic Health Science Centre, University of Manchester, Manchester, UK

^7^Diabetes, Endocrinology and Metabolism Centre, NIHR Manchester Biomedical Research Centre, Manchester University NHS Foundation Trust, Manchester, UK

^8^Department of Epidemiology, Human Genetics and Environmental Sciences, School of Public Health, The University of Texas Health Science Center at Houston, Houston, TX, United States

^9^Center for Spatial-temporal Modeling for Applications in Population Sciences, School of Public Health, The University of Texas Health Science Center at Houston, Houston, TX, United States

*Authors contributed equally to this manuscript.

**Corresponding Author:** Andrew J. K. Phillips

andrew.phillips@monash.edu

**Table of Contents**

**Table S1.** UK Biobank study protocol documents 3

**Table S2.** UK Biobank covariates 4

**Table S3.** Covariates included in statistical analyses 7

**Figure S4.** Methodology overview 8

**Table S5.** Missing data across covariates 9

**Table S6.** Model 3 adjusted for cardiometabolic health, sleep duration, mental health, photoperiod, chronotype, and exclusion of shift workers 10

**Table S7.** Models 1-3 adjusted for night-light*sex interaction 13

**Table S8.** Risk of type 2 diabetes after excluding pre-diabetes 14

**Table S9.** Competing-risk of incident type 2 diabetes against risk of participant mortality 15

**Figure S10.** Risk of type 2 diabetes for light exposures across twenty-four-hours. 16

**Table S11.** Proportional hazards assumption test for Models 1-3 17

**S12.** Supplementary Methods 18

**S13.** STROBE Statement 22

# **Table S1. UK Biobank study protocol documents**

| **Protocol document** | **Link** |
| --- | --- |
| Ethical approval | https://www.ukbiobank.ac.uk/learn-more-about-uk-biobank/about-us/ethics |
| Study participation invitation | https://biobank.ctsu.ox.ac.uk/crystal/refer.cgi?id=100253 |
| First occurrence of health outcomes | https://biobank.ndph.ox.ac.uk/showcase/refer.cgi?id=593 |
| Death registry | https://biobank.ctsu.ox.ac.uk/crystal/refer.cgi?id=115559 |
| Axivity AX3: Participant instructions | https://biobank.ndph.ox.ac.uk/showcase/refer.cgi?id=141141 |
| Axivity AX3: Collection and processing | https://biobank.ndph.ox.ac.uk/showcase/refer.cgi?id=131600 |
| Assessment centre: Reception and consent | https://biobank.ndph.ox.ac.uk/showcase/refer.cgi?id=100230 |
|  | https://biobank.ndph.ox.ac.uk/showcase/ukb/docs/Reception.pdf |
| Assessment centre: Physical measurements | https://biobank.ndph.ox.ac.uk/showcase/refer.cgi?id=100225 |
|  | https://biobank.ndph.ox.ac.uk/showcase/refer.cgi?id=5636 |
|  | https://biobank.ndph.ox.ac.uk/showcase/refer.cgi?id=1227 |

# **Table S2. UK Biobank covariates**

| **Variable (UKB ID)** | **Collection** | **Description** | **Link** |
| --- | --- | --- | --- |
| Age (21003) | Registry | Participant age at assessment centre visit, in years, obtained from NHS Primary Care Trust registries. Confirmed with participants at assessment centre. | https://biobank.ndph.ox.ac.uk/showcase/field.cgi?id=21003 |
| Sex (31) | Registry | From NHS Primary Care Trust registries. Confirmed with participants at assessment centre. | https://biobank.ndph.ox.ac.uk/showcase/field.cgi?id=31 |
| Ethnic background (21000) | Assessment centre visit: Questionnaire | Ethnic group (white, mixed, Asian/Asian British, black/black British, Chinese, other, PNTA) and ethnic background sub-categories. | https://biobank.ndph.ox.ac.uk/showcase/field.cgi?id=21000 |
| Qualifications (6138) | Assessment centre visit: Questionnaire | University, A Levels, O Levels, CSE, NVQ/HND/HNC, other, none, PNTA | https://biobank.ndph.ox.ac.uk/showcase/field.cgi?id=6138 |
| Current employment status (6142) | Assessment centre visit: Questionnaire | Paid employment, unemployed, retired, home/family caretaker, unable to work, volunteer, student, other | https://biobank.ndph.ox.ac.uk/showcase/field.cgi?id=6142 |
| Average total household income before tax (738) | Assessment centre visit: Questionnaire | Income brackets: <£18,000, £18,000-£29,900, £30,000-£51,900, £52,000-£100,000, >£100,000, DNK, PNTA | https://biobank.ndph.ox.ac.uk/showcase/field.cgi?id=738 |
| Townsend deprivation index (189) | Registry | Scores represent deprivation by local area, quantified by average home ownership, car ownership, household overcrowding, and employment rate, and were derived using national census data at time of recruitment. | https://biobank.ndph.ox.ac.uk/showcase/field.cgi?id=189 |
| Smoking status (20116) | Assessment centre visit: Questionnaire | Smoking status / history: never, previous, current, PNTA | https://biobank.ndph.ox.ac.uk/showcase/field.cgi?id=20116 |
| Alcohol intake frequency (1558) | Assessment centre visit: Questionnaire | Daily, 3-4 times per week, 1-2 times per week, 1-3 times per month, special occasions only, never, PNTA | https://biobank.ndph.ox.ac.uk/showcase/field.cgi?id=1558 |
| Urbanicity (20118) | Registry | ‘Urban’ (population ≥ 10,000) and ‘non-urban’ (population < 10,000), defined according to population density of participant’s local area. Derived from the UK Office for National Statistics. | https://biobank.ndph.ox.ac.uk/showcase/field.cgi?id=20118 |
| Shift work (826) | Assessment centre visit: Questionnaire | 'Does your work involve shift work?' Answers: never, sometimes, usually, always, DNK, PNTA | https://biobank.ndph.ox.ac.uk/showcase/field.cgi?id=826 |
| Night shift work (3426) | Assessment centre visit: Questionnaire | 'Does your work involve night shifts?' Answers: never, sometimes, usually, always, DNK, PNTA | https://biobank.ndph.ox.ac.uk/showcase/field.cgi?id=3426 |
| Body mass index (21001) | Assessment centre visit: Physical measurements | Weight (kg) / height (m)^2 | https://biobank.ndph.ox.ac.uk/showcase/field.cgi?id=21001 |
| High density lipoprotein (30760) | Assessment centre visit: Physical measurements | Blood biochemistry assay | https://biobank.ndph.ox.ac.uk/showcase/field.cgi?id=30760 |
| Low density lipoprotein (30780) | Assessment centre visit: Physical measurements | Blood biochemistry assay | https://biobank.ndph.ox.ac.uk/showcase/field.cgi?id=30780 |
| Triglycerides (30870) | Assessment centre visit: Physical measurements | Blood biochemistry assay | https://biobank.ndph.ox.ac.uk/showcase/field.cgi?id=30870 |
| Systolic blood pressure (4080) | Assessment centre visit: Physical measurements | Two readings taken at the start and end of assessment centre visit | https://biobank.ndph.ox.ac.uk/showcase/field.cgi?id=4080 |
| Diastolic blood pressure (4079) | Assessment centre visit: Physical measurements | Two readings taken at the start and end of assessment centre visit | https://biobank.ndph.ox.ac.uk/showcase/field.cgi?id=4079 |
| Glycated haemoglobin (HbA1c) (30750) | Assessment centre visit: Physical measurements | Blood biochemistry assay | https://biobank.ndph.ox.ac.uk/showcase/field.cgi?id=30750 |
| Glucose (30740) | Assessment centre visit: Physical measurements | Blood biochemistry assay | https://biobank.ndph.ox.ac.uk/showcase/field.cgi?id=30740 |
| Physical activity (90012) | Accelerometer | Device average acceleration across one week data collection | https://biobank.ndph.ox.ac.uk/showcase/field.cgi?id=90012 |
| Frequency of depressed mood (2050) | Assessment centre visit: Questionnaire | ‘Over the past two weeks, how often have you felt down, depressed, and hopeless?’ Answers: not at all, several days, more than half the days, nearly every day, DNK, PNTA | https://biobank.ndph.ox.ac.uk/showcase/field.cgi?id=2050 |
| Frequency of unenthusiasm / disinterest (2060) | Assessment centre visit: Questionnaire | ‘Over the past two weeks, how often have you had little interest or pleasure in doing things?’ Answers: not at all, several days, more than half the days, nearly every day, DNK, PNTA | https://biobank.ndph.ox.ac.uk/showcase/field.cgi?id=2060 |
| Frequency of tenseness / restlessness (2070) | Assessment centre visit: Questionnaire | ‘Over the past two weeks, how often have you felt tense, fidgety, or restless?’ Answers: not at all, several days, more than half the days, nearly every day, DNK, PNTA | https://biobank.ndph.ox.ac.uk/showcase/field.cgi?id=2070 |
| Frequency of tiredness / lethargy (2080) | Assessment centre visit: Questionnaire | ‘Over the past two weeks, how often have you felt tired or had little energy?’ Answers: not at all, several days, more than half the days, nearly every day, DNK, PNTA | https://biobank.ndph.ox.ac.uk/showcase/field.cgi?id=2080 |
| Visited GP for mental health (2090) | Assessment centre visit: Questionnaire | 'Have you ever seen a general practitioner (GP) for nerves, anxiety, tension, or depression?’ Answers: yes, no, DNK, PNTA | https://biobank.ndph.ox.ac.uk/showcase/field.cgi?id=2090 |
| Visited psychiatrist for mental health (2100) | Assessment centre visit: Questionnaire | 'Have you ever seen a psychiatrist for nerves, anxiety, tension, or depression?’ Answers: yes, no, DNK, PNTA | https://biobank.ndph.ox.ac.uk/showcase/field.cgi?id=2100 |
| Chronotype (1180) | Assessment centre visit: Questionnaire | ‘Do you consider yourself to be?’ Answers: definitely a ‘morning’ person, more a ‘morning’ than ‘evening’ person, more an ‘evening’ than ‘morning’ person, definitely an ‘evening’ person, DNK | https://biobank.ndph.ox.ac.uk/showcase/field.cgi?id=1180 |

DNK = ‘do not know’; PNTA = ‘prefer not to answer’

# **Table S3. Covariates included in statistical analyses**

| **Covariate** | **UKB ID(s)** | **Description** |
| --- | --- | --- |
| Age | 21003 | Continuous, included as age at commencement of light / activity recording |
| Sex | 31 | Binary, male = 1, female = 0 |
| Ethnicity | 21000 | Binary, white ethnic group = 1, other ethnic group = 0 |
| Education | 6138 | Categorical: ‘University’, ‘other non-university’ (selection of any education category except ‘University’), ‘none’ (referent) |
| Employment status | 6142 | Binary, employed = 1, other categories = 0 |
| Income | 738 | Categorical: ‘<£18k’ (referent), ‘£18k-£29·9k’, ‘£30k-£51·9k’, ‘£52k-£100k’, ‘>£100k’, and ‘Unknown’ |
| Deprivation | 189 | Continuous, included as recorded |
| Physical activity | 90012 | Continuous, included as recorded |
| Smoking status | 20116 | Categorical: ‘current’, ‘previous’, 'never' (referent) |
| Alcohol consumption | 1558 | Continuous, days per week consuming alcohol. ‘Daily’ = 7, ‘3-4 times per week’ = 3·5, ‘1-2 times per week’ = 1·5, ‘1-3 times per month’ = 2*12/365·25*7, ‘special occasions only’ = 1*12/365·25*7, ‘never’ = 0 |
| Healthy diet | 1289, 1299, 1309, 1319, 1329, 1339, 1349, 1359, 1369, 1379, 1389, 1408, 1418, 1428, 1438, 1448, 1458, 1468  2654, 3680, 6144 | Binary: healthy = 1, unhealthy = 0. Calculated across 10 nutritional intake criteria for cardiometabolic health, as reported previously.^1^ Classified as healthy diet if ≥5 out of 10 criteria were met. |
| Urbanicity | 20118 | Binary: urban = 1, rural = 0 |
| Shift work | 826, 3426, 6142 | Binary: shift-worker = 1, non-shift worker = 0. ‘Sometimes’, ‘usually’ or 'always' for either ‘shift work’ or ‘night shift work’ = 1, ‘never/rarely’ = 0. Participants coded as ‘unemployed’ were assigned as non-shift workers, according to the derived ‘employment status’ variable. |
| BMI | 21001 | Binary: BMI > 30 = 1, BMI ≤ 30 = 0 |
| Cholesterol ratio | 30760, 30780, 30870 | Binary: ‘high’ = cholesterol ratio > 3·75 for males or > 3·00 for females. Calculated as cholesterol ratio = (HDL + LDL + 0·2*triglycerides)/HDL |
| Hypertension | 4080, 4079 | Binary: ‘high’ = systolic > 140 or diastolic > 90, where systolic and diastolic were included as the average of two readings (mmHg) |
| HbA1c | 30750 | Binary: ≥39 mmol/mol = 1, <39 mmol/mol = 0 |
| Random glucose | 30740 | Binary: ≥7.8 mmol/L = 1, <7.8 mmol/L = 0 |
| Depressed mood | 2050 | Binary: ‘not at all’ = 0, ‘several days’, ‘more than half the days’, or ‘nearly every day’ = 1 |
| Unenthusiasm / disinterest | 2060 | Binary: ‘not at all’ = 0, ‘several days’, ‘more than half the days’, or ‘nearly every day’ = 1 |
| Tenseness / restlessness | 2070 | Binary: ‘not at all’ = 0, ‘several days’, ‘more than half the days’, or ‘nearly every day’ = 1 |
| Tiredness / lethargy | 2080 | Binary: ‘not at all’ = 0, ‘several days’, ‘more than half the days’, or ‘nearly every day’ = 1 |
| Visited GP for mental health | 2090 | Binary: yes = 1, no = 0 |
| Visited psychiatrist for mental health | 2100 | Binary: yes = 1, no = 0 |
| Chronotype | 1180 | Categorical: ‘mostly morning’ (referent), ‘definitely morning’, ‘mostly evening’, ‘definitely evening’ |
| Photoperiod | - | Continuous, calculated from date of light recording and coordinates of 53.4808° N, 2.2426° W (Manchester), using ‘getSunlightTimes()’ in the ‘suncalc’ package in R. |

**
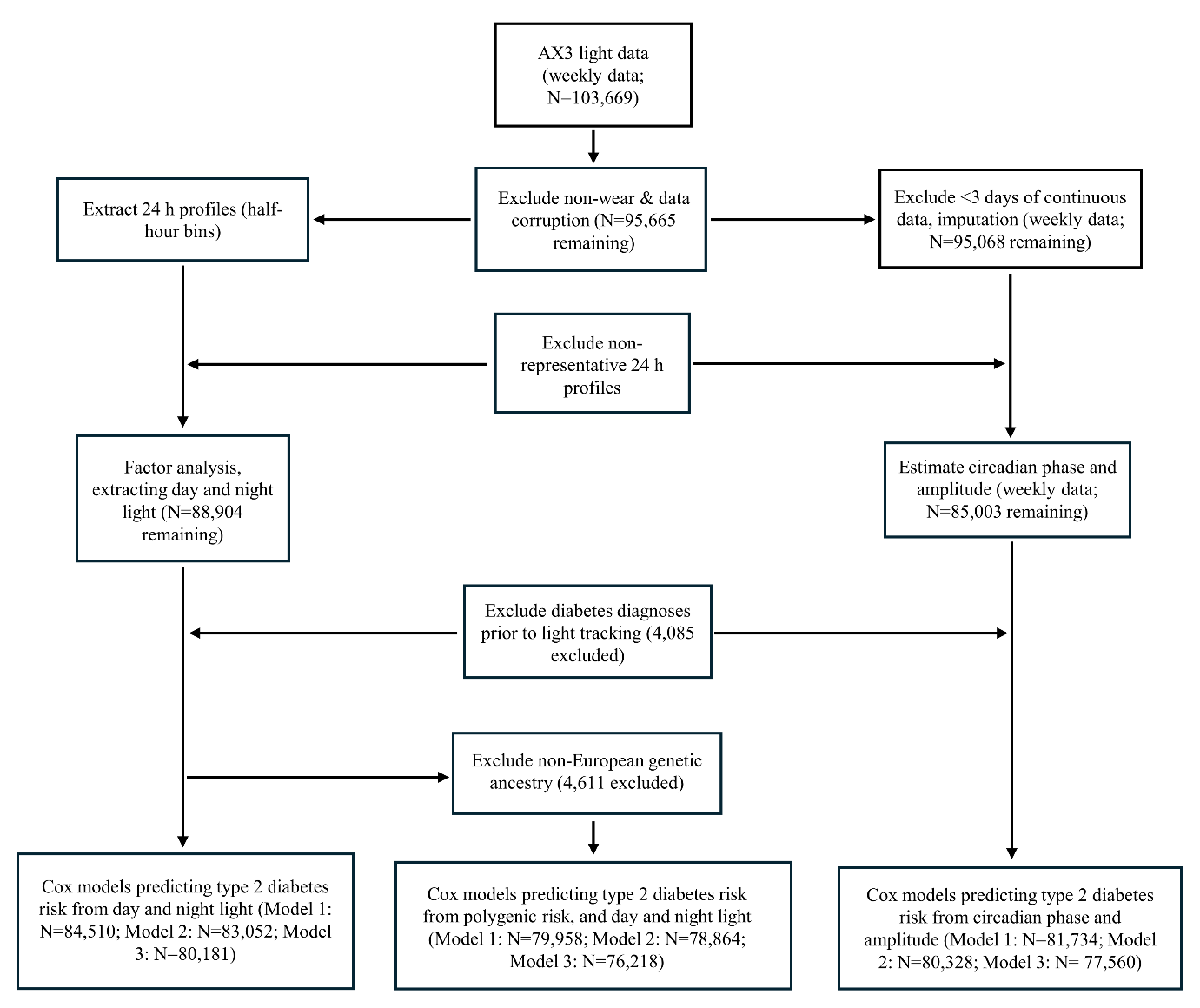
**

**Figure S4. Methodology overview.** Data processing and exclusion steps between light recording and final analyses.

# **Table S5. Missing data across covariates**

| **Covariate** | **Missing, N (%)** |
| --- | --- |
| Sex | 0 (0) |
| Age | 0 (0) |
| Alcohol | 68 (0∙08) |
| Deprivation | 101 (0∙12) |
| Physical activity | 135 (0∙16) |
| Smoking | 219 (0∙26) |
| Ethnicity | 280 (0∙33) |
| Income | 572 (0∙67) |
| Employment | 579 (0∙68) |
| Urbanicity | 821 (0∙97) |
| Education | 843 (0∙99) |
| Diet | 2340 (2∙8) |

Percentages were calculated from missing data for each covariate within 84,790 participants with complete light data and no diabetes diagnosis prior to light recording.

# **Table S6. Model 3 adjusted for cardiometabolic health, sleep duration, mental health, photoperiod, chronotype, and exclusion of shift workers**

|  |  | **Percentile** | **Cases % (N)** | **HR [95% CI]** | **p-value** |
| --- | --- | --- | --- | --- | --- |
| Model 3 + BMI | Night | 0-50% (ref.) | 1∙95 (780) | ∙∙ | ∙∙ |
| N = 80,034 |  | 50-70% | 2∙46 (393) | 1∙24 [1∙09-1∙40]* | 0∙00068 |
|  |  | 70-90% | 2∙67 (428) | 1∙30 [1∙16-1∙47]* | <0∙0001 |
|  |  | 90-100% | 3∙15 (252) | 1∙37 [1∙19-1∙59]* | <0∙0001 |
|  | Day | 0-50% (ref.) | 2∙34 (937) | ∙∙ | ∙∙ |
|  |  | 50-70% | 2∙38 (381) | 1∙04 [0∙93-1∙18] | 0∙49 |
|  |  | 70-90% | 2∙25 (360) | 1∙00 [0∙88-1∙13] | 0∙96 |
|  |  | 90-100% | 2∙19 (175) | 0∙95 [0∙80-1∙12] | 0∙52 |
| Model 3 + hypertension | Night | 0-50% (ref.) | 1∙97 (751) | ∙∙ | ∙∙ |
| N = 76,281 |  | 50-70% | 2∙47 (377) | 1∙30 [1∙15-1∙47]* | <0∙0001 |
|  |  | 70-90% | 2∙70 (412) | 1∙39 [1∙23-1∙57]* | <0∙0001 |
|  |  | 90-100% | 3∙20 (244) | 1∙55 [1∙34-1∙80]* | <0∙0001 |
|  | Day | 0-50% (ref.) | 2∙35 (898) | ∙∙ | ∙∙ |
|  |  | 50-70% | 2∙42 (369) | 1∙07 [0∙95-1∙21] | 0∙29 |
|  |  | 70-90% | 2∙29 (350) | 1∙02 [0∙90-1∙16] | 0∙77 |
|  |  | 90-100% | 2∙19 (167) | 0∙95 [0∙80-1∙13] | 0∙57 |
| Model 3 + cholesterol ratio | Night | 0-50% (ref.) | 1∙98 (688) | ∙∙ | ∙∙ |
| N = 69,414 |  | 50-70% | 2∙44 (339) | 1∙26 [1∙11-1∙44]* | 0∙00051 |
|  |  | 70-90% | 2∙70 (375) | 1∙39 [1∙22-1∙57]* | <0∙0001 |
|  |  | 90-100% | 3∙10 (215) | 1∙47 [1∙26-1∙72]* | <0∙0001 |
|  | Day | 0-50% (ref.) | 2∙36 (818) | ∙∙ | ∙∙ |
|  |  | 50-70% | 2∙42 (336) | 1∙06 [0∙94-1∙21] | 0∙35 |
|  |  | 70-90% | 2∙18 (303) | 0∙97 [0∙85-1∙11] | 0∙64 |
|  |  | 90-100% | 2∙31 (160) | 1∙00 [0∙84-1∙19] | 0∙99 |
| Model 3 + sleep duration | Night | 0-50% (ref.) | 1∙95 (751) | ∙∙ | ∙∙ |
| N = 77,166 |  | 50-70% | 2∙46 (380) | 1∙25 [1∙10-1∙41]* | 0∙00056 |
|  |  | 70-90% | 2∙73 (421) | 1∙32 [1∙17-1∙50]* | <0∙0001 |
|  |  | 90-100% | 3∙20 (247) | 1∙39 [1∙20-1∙62]* | <0∙0001 |
|  | Day | 0-50% (ref.) | 2∙35 (907) | ∙∙ | ∙∙ |
|  |  | 50-70% | 2∙42 (373) | 1∙07 [0∙95-1∙21] | 0∙27 |
|  |  | 70-90% | 2∙24 (346) | 1∙01 [0∙89-1∙15] | 0∙82 |
|  |  | 90-100% | 2∙24 (173) | 1∙01 [0∙85-1∙19] | 0∙91 |
| Model 3 + depressed mood | Night | 0-50% (ref.) | 1∙94 (758) | ∙∙ | ∙∙ |
| N = 77,975 |  | 50-70% | 2∙43 (379) | 1∙28 [1∙13-1∙45]* | <0∙0001 |
|  |  | 70-90% | 2∙63 (410) | 1∙37 [1∙21-1∙54]* | <0∙0001 |
|  |  | 90-100% | 3∙13 (244) | 1∙51 [1∙31-1∙75]* | <0∙0001 |
|  | Day | 0-50% (ref.) | 2∙31 (899) | ∙∙ | ∙∙ |
|  |  | 50-70% | 2∙41 (376) | 1∙09 [0∙97-1∙23] | 0∙17 |
|  |  | 70-90% | 2∙21 (344) | 1∙02 [0∙89-1∙15] | 0∙81 |
|  |  | 90-100% | 2∙21 (172) | 1∙01 [0∙85-1∙19] | 0∙93 |
| Model 3 + unenthusiasm / disinterest | Night | 0-50% (ref.) | 1∙95 (766) | ∙∙ | ∙∙ |
| N = 78,651 |  | 50-70% | 2∙46 (387) | 1∙29 [1∙14-1∙46]* | <0∙0001 |
|  |  | 70-90% | 2∙64 (416) | 1∙37 [1∙22-1∙55]* | <0∙0001 |
|  |  | 90-100% | 3∙14 (247) | 1∙53 [1∙32-1∙77]* | <0∙0001 |
|  | Day | 0-50% (ref.) | 2∙32 (913) | ∙∙ | ∙∙ |
|  |  | 50-70% | 2∙40 (377) | 1∙08 [0∙95-1∙21] | 0∙24 |
|  |  | 70-90% | 2∙24 (352) | 1∙02 [0∙90-1∙15] | 0∙78 |
|  |  | 90-100% | 2∙21 (174) | 1∙00 [0∙84-1∙18] | 0∙95 |
| Model 3 + tenseness / restlessness | Night | 0-50% (ref.) | 1∙93 (756) | ∙∙ | ∙∙ |
| N = 78,260 |  | 50-70% | 2∙45 (384) | 1∙30 [1∙15-1∙47]* | <0∙0001 |
|  |  | 70-90% | 2∙66 (417) | 1∙40 [1∙24-1∙58]* | <0∙0001 |
|  |  | 90-100% | 3∙14 (246) | 1∙54 [1∙33-1∙79]* | <0∙0001 |
|  | Day | 0-50% (ref.) | 2∙32 (908) | ∙∙ | ∙∙ |
|  |  | 50-70% | 2∙37 (371) | 1∙06 [0∙94-1∙20] | 0∙33 |
|  |  | 70-90% | 2∙24 (351) | 1∙02 [0∙90-1∙15] | 0∙77 |
|  |  | 90-100% | 2∙21 (173) | 0∙99 [0∙84-1∙18] | 0∙94 |
| Model 3 + tiredness / lethargy | Night | 0-50% (ref.) | 1∙95 (767) | ∙∙ | ∙∙ |
| N = 78,677 |  | 50-70% | 2∙45 (386) | 1∙28 [1∙14-1∙45]* | <0∙0001 |
|  |  | 70-90% | 2∙66 (419) | 1∙37 [1∙22-1∙55]* | <0∙0001 |
|  |  | 90-100% | 3∙18 (250) | 1∙52 [1∙32-1∙76]* | <0∙0001 |
|  | Day | 0-50% (ref.) | 2∙32 (913) | ∙∙ | ∙∙ |
|  |  | 50-70% | 2∙42 (381) | 1∙09 [0∙96-1∙23] | 0∙17 |
|  |  | 70-90% | 2∙24 (353) | 1∙03 [0∙91-1∙16] | 0∙66 |
|  |  | 90-100% | 2∙22 (175) | 1∙01 [0∙86-1∙20] | 0∙87 |
| Model 3 + visited GP for mental health | Night | 0-50% (ref.) | 1∙96 (783) | ∙∙ | ∙∙ |
| N = 79,831 |  | 50-70% | 2∙46 (393) | 1∙29 [1∙14-1∙45]* | <0∙0001 |
|  |  | 70-90% | 2∙67 (427) | 1∙38 [1∙22-1∙55]* | <0∙0001 |
|  |  | 90-100% | 3∙13 (250) | 1∙50 [1∙30-1∙74]* | <0∙0001 |
|  | Day | 0-50% (ref.) | 2∙34 (936) | ∙∙ | ∙∙ |
|  |  | 50-70% | 2∙39 (382) | 1∙07 [0∙95-1∙21] | 0∙26 |
|  |  | 70-90% | 2∙26 (361) | 1∙02 [0∙91-1∙16] | 0∙70 |
|  |  | 90-100% | 2∙18 (174) | 0∙99 [0∙84-1∙17] | 0∙87 |
| Model 3 + visited psychiatrist for mental health | Night | 0-50% (ref.) | 1∙96 (785) | ∙∙ | ∙∙ |
| N = 79,979 |  | 50-70% | 2∙46 (394) | 1∙29 [1∙14-1∙46]* | <0∙0001 |
|  |  | 70-90% | 2∙69 (431) | 1∙39 [1∙24-1∙57]* | <0∙0001 |
|  |  | 90-100% | 3∙15 (252) | 1∙52 [1∙32-1∙76]* | <0∙0001 |
|  | Day | 0-50% (ref.) | 2∙35 (941) | ∙∙ | <0∙0001 |
|  |  | 50-70% | 2∙40 (384) | 1∙07 [0∙95-1∙20] | 0∙29 |
|  |  | 70-90% | 2∙26 (362) | 1∙02 [0∙90-1∙15] | 0∙75 |
|  |  | 90-100% | 2∙19 (175) | 0∙98 [0∙83-1∙16] | 0∙81 |
| Model 3 + chronotype | Night | 0-50% (ref.) | 1∙97 (710) | ∙∙ | ∙∙ |
| N = 72,101 |  | 50-70% | 2∙50 (360) | 1∙30 [1∙15-1∙48]* | <0∙0001 |
|  |  | 70-90% | 2∙73 (393) | 1∙39 [1∙23-1∙58]* | <0∙0001 |
|  |  | 90-100% | 3∙26 (235) | 1∙54 [1∙32-1∙80]* | <0∙0001 |
|  | Day | 0-50% (ref.) | 2∙37 (855) | ∙∙ | ∙∙ |
|  |  | 50-70% | 2∙45 (353) | 1∙07 [0∙94-1∙21] | 0∙30 |
|  |  | 70-90% | 2∙29 (330) | 1∙01 [0∙89-1∙15] | 0∙88 |
|  |  | 90-100% | 2∙22 (160) | 0∙98 [0∙82-1∙17] | 0∙82 |
| Model 3 + photoperiod | Night | 0-50% (ref.) | 1∙96 (786) | ∙∙ | ∙∙ |
| N = 80,181 |  | 50-70% | 2∙46 (394) | 1∙29 [1∙14-1∙45]* | <0∙0001 |
|  |  | 70-90% | 2∙69 (431) | 1∙39 [1∙24-1∙57]* | <0∙0001 |
|  |  | 90-100% | 3∙16 (253) | 1∙53 [1∙33-1∙77]* | <0∙0001 |
|  | Day | 0-50% (ref.) | 2∙35 (942) | ∙∙ | ∙∙ |
|  |  | 50-70% | 2∙39 (384) | 1∙06 [0∙94-1∙20] | 0∙36 |
|  |  | 70-90% | 2∙26 (362) | 1∙01 [0∙88-1∙17] | 0∙84 |
|  |  | 90-100% | 2∙20 (176) | 0∙98 [0∙81-1∙18] | 0∙8 |
| Model 3, excluding shift workers | Night | 0-50% (ref.) | 1∙98 (730) | ∙∙ | ∙∙ |
| N = 73,761 |  | 50-70% | 2∙43 (358) | 1∙30 [1∙14-1∙47]* | <0∙0001 |
|  |  | 70-90% | 2∙54 (374) | 1∙35 [1∙19-1∙53]* | <0∙0001 |
|  |  | 90-100% | 3∙12 (230) | 1∙57 [1∙35-1∙82]* | <0∙0001 |
|  | Day | 0-50% (ref.) | 2∙29 (846) | ∙∙ | ∙∙ |
|  |  | 50-70% | 2∙35 (347) | 1∙07 [0∙94-1∙21] | 0∙30 |
|  |  | 70-90% | 2∙28 (337) | 1∙04 [0∙92-1∙18] | 0∙54 |
|  |  | 90-100% | 2∙20 (162) | 0∙98 [0∙82-1∙16] | 0∙78 |

Data are proportional hazards (95% CI) of type 2 diabetes. Model 3 covariates: age, sex, ethnicity, income, material deprivation, education, employment status, smoking status, alcohol consumption, healthy diet, physical activity, and urbanicity. * p<·05.

# **Table S7. Models 1-3 adjusted for night-light*sex interaction**

|  |  |  | **Male** |  |  | **Female** |  |  | **Interaction** |  |
| --- | --- | --- | --- | --- | --- | --- | --- | --- | --- | --- |
|  |  | **Percentile** | **Cases % (N)** | **HR [95% CI]** | **p-value** | **Cases % (N)** | **HR [95% CI]** | **p-value** | **Estimate (SE)** | **p-value** |
| Model 1 | Night | 0-50% (ref.) | 2∙71 (483) | ∙∙ | ∙∙ | 1∙44 (352) | ∙∙ | <0∙0001 | ∙∙ | ∙∙ |
| N = 84,510 |  | 50-70% | 3∙68 (263) | 1∙35 [1∙16-1∙57]* | 0∙00011 | 1∙67 (163) | 1∙29 [1∙07-1∙56]* | 0∙0068 | -0∙041 (0∙12) | 0∙73 |
|  |  | 70-90% | 3∙78 (270) | 1∙41 [1∙21-1∙64]* | <0∙0001 | 1∙94 (189) | 1∙49 [1∙25-1∙78]* | <0∙0001 | 0∙057 (0∙12) | 0∙63 |
|  |  | 90-100% | 4∙23 (151) | 1∙57 [1∙31-1∙89]* | <0∙0001 | 2∙38 (116) | 1∙81 [1∙46-2∙23]* | <0∙0001 | 0∙14 (0∙14) | 0∙33 |
|  | Day | 0-50% (ref.) | 3∙36 (599) | ∙∙ | ∙∙ | 1∙71 (418) | ∙∙ | ∙∙ | ∙∙ | ∙∙ |
|  |  | 50-70% | 3∙24 (231) | 0∙97 [0∙86-1∙09] | 0∙59 | 1∙83 (179) | 0∙97 [0∙86-1∙09] | 0∙59 | ∙∙ | ∙∙ |
|  |  | 70-90% | 2∙98 (213) | 0∙84 [0∙75-0∙95]* | 0∙0055 | 1∙66 (162) | 0∙84 [0∙75-0∙95]* | 0∙0055 | ∙∙ | ∙∙ |
|  |  | 90-100% | 3∙47 (124) | 0∙73 [0∙62-0∙86]* | 0∙00010 | 1∙25 (61) | 0∙73 [0∙62-0∙86]* | 0∙00010 | ∙∙ | ∙∙ |
| Model 2 | Night | 0-50% (ref.) | 2∙68 (471) | ∙∙ | ∙∙ | 1∙45 (347) | ∙∙ | ∙∙ | ∙∙ | ∙∙ |
| N = 83,052 |  | 50-70% | 3∙60 (253) | 1∙30 [1∙11-1∙51]* | 0∙00085 | 1∙65 (158) | 1∙25 [1∙04-1∙51]* | 0∙02 | -0∙037 (0∙12) | 0∙76 |
|  |  | 70-90% | 3∙73 (262) | 1∙37 [1∙18-1∙60]* | <0∙0001 | 1∙95 (187) | 1∙48 [1∙24-1∙77]* | <0∙0001 | 0∙073 (0∙12) | 0∙54 |
|  |  | 90-100% | 4∙25 (149) | 1∙52 [1∙26-1∙84]* | <0∙0001 | 2∙34 (112) | 1∙71 [1∙38-2∙12]* | <0∙0001 | 0∙11 (0∙14) | 0∙43 |
|  | Day | 0-50% (ref.) | 3∙29 (578) | ∙∙ | ∙∙ | 1∙71 (409) | ∙∙ | ∙∙ | ∙∙ | ∙∙ |
|  |  | 50-70% | 3∙22 (226) | 0∙99 [0∙88-1∙11] | 0∙83 | 1∙81 (174) | 0∙99 [0∙88-1∙11] | 0∙83 | ∙∙ | ∙∙ |
|  |  | 70-90% | 2∙98 (209) | 0∙88 [0∙78-0∙99]* | 0∙037 | 1∙69 (162) | 0∙88 [0∙78-0∙99]* | 0∙037 | ∙∙ | ∙∙ |
|  |  | 90-100% | 3∙48 (122) | 0∙77 [0∙65-0∙90]* | 0∙0015 | 1∙23 (59) | 0∙77 [0∙65-0∙90]* | 0∙0015 | ∙∙ | ∙∙ |
| Model 3 | Night | 0-50% (ref.) | 2∙67 (455) | ∙∙ | ∙∙ | 1∙44 (331) | ∙∙ | ∙∙ | ∙∙ | ∙∙ |
| N = 80,181 |  | 50-70% | 3∙58 (244) | 1∙31 [1∙12-1∙53]* | 0∙00069 | 1∙63 (150) | 1∙25 [1∙03-1∙52]* | 0∙022 | -0∙045 (0∙13) | 0∙72 |
|  |  | 70-90% | 3∙76 (256) | 1∙37 [1∙17-1∙60]* | <0∙0001 | 1∙9 (175) | 1∙43 [1∙19-1∙72]* | 0∙00014 | 0∙045 (0∙12) | 0∙71 |
|  |  | 90-100% | 4∙26 (145) | 1∙48 [1∙22-1∙78]* | <0∙0001 | 2∙34 (108) | 1∙61 [1∙30-2∙01]* | <0∙0001 | 0∙089 (0∙15) | 0∙54 |
|  | Day | 0-50% (ref.) | 3∙26 (556) | ∙∙ | ∙∙ | 1∙67 (386) | ∙∙ | ∙∙ | ∙∙ | ∙∙ |
|  |  | 50-70% | 3∙21 (219) | 1∙06 [0∙94-1∙20] | 0∙32 | 1∙79 (165) | 1∙06 [0∙94-1∙20] | 0∙32 | ∙∙ | ∙∙ |
|  |  | 70-90% | 2∙99 (204) | 1∙02 [0∙90-1∙15] | 0∙78 | 1∙71 (158) | 1∙02 [0∙90-1∙15] | 0∙78 | ∙∙ | ∙∙ |
|  |  | 90-100% | 3∙55 (121) | 0∙98 [0∙83-1∙16] | 0∙82 | 1∙19 (55) | 0∙98 [0∙83-1∙16] | 0∙82 | ∙∙ | ∙∙ |

Data are proportional hazards (95% CI) of type 2 diabetes for males and females. All models include a night light*sex interaction term. Models under ‘Male’ include sex as male=0, female=1 and models under ‘Female’ include sex as female=0, male=1. Estimates and p-values for interaction terms are common to both ‘Male’ and ‘Female’ models. Model covariates: model 1: age, sex, and ethnicity; model 2: model 1 covariates plus income, material deprivation, education, and employment status; and model 3: model 2 covariates plus smoking status, alcohol consumption, healthy diet, physical activity, and urbanicity. * p<·05.

# **Table S8. Risk of type 2 diabetes after excluding pre-diabetes**

|  |  | **Percentile** | **Cases % (N)** | **HR [95% CI]** | **p-value** |
| --- | --- | --- | --- | --- | --- |
| Model 1 | Night | 0-50% (ref.) | 1∙05 (387) | ∙∙ | ∙∙ |
| N = 73,677 |  | 50-70% | 1∙32 (194) | 1∙29 [1∙08-1∙53]* | 0∙0041 |
|  |  | 70-90% | 1∙33 (196) | 1∙33 [1∙12-1∙58]* | 0∙0014 |
|  |  | 90-100% | 1∙48 (109) | 1∙49 [1∙20-1∙85]* | 0∙00027 |
|  | Day | 0-50% (ref.) | 1∙22 (451) | ∙∙ | ∙∙ |
|  |  | 50-70% | 1∙17 (173) | 0∙93 [0∙78-1∙11] | 0∙42 |
|  |  | 70-90% | 1∙25 (184) | 0∙95 [0∙80-1∙13] | 0∙55 |
|  |  | 90-100% | 1∙06 (78) | 0∙71 [0∙56-0∙91]* | 0∙0061 |
| Model 2 | Night | 0-50% (ref.) | 1∙05 (383) | ∙∙ | ∙∙ |
| N = 72827 |  | 50-70% | 1∙28 (187) | 1∙23 [1∙03-1∙47]* | 0∙02 |
|  |  | 70-90% | 1∙31 (191) | 1∙29 [1∙08-1∙54]* | 0∙0048 |
|  |  | 90-100% | 1∙48 (108) | 1∙43 [1∙15-1∙78]* | 0∙0012 |
|  | Day | 0-50% (ref.) | 1∙21 (439) | ∙∙ | ∙∙ |
|  |  | 50-70% | 1∙17 (171) | 0∙96 [0∙80-1∙14] | 0∙61 |
|  |  | 70-90% | 1∙26 (184) | 1∙00 [0∙84-1∙19] | 0∙97 |
|  |  | 90-100% | 1∙03 (75) | 0∙74 [0∙58-0∙95]* | 0∙017 |
| Model 3 | Night | 0-50% (ref.) | 1∙05 (370) | ∙∙ | ∙∙ |
| N = 70,311 |  | 50-70% | 1∙28 (180) | 1∙25 [1∙04-1∙50]* | 0∙015 |
|  |  | 70-90% | 1∙33 (187) | 1∙30 [1∙08-1∙55]* | 0∙0045 |
|  |  | 90-100% | 1∙48 (104) | 1∙35 [1∙08-1∙69]* | 0∙0077 |
|  | Day | 0-50% (ref.) | 1∙2 (422) | ∙∙ | ∙∙ |
|  |  | 50-70% | 1∙18 (166) | 1∙04 [0∙87-1∙25] | 0∙67 |
|  |  | 70-90% | 1∙29 (181) | 1∙18 [0∙99-1∙41] | 0∙072 |
|  |  | 90-100% | 1∙02 (72) | 0∙97 [0∙75-1∙25] | 0∙81 |

Data are proportional hazards (95% CI) of type 2 diabetes∙ Model covariates: model 1: age, sex, and ethnicity; model 2: model 1 covariates plus income, material deprivation, education, and employment status; and model 3: model 2 covariates plus smoking status, alcohol consumption, healthy diet, physical activity, and urbanicity. * p<·05.

# **Table S9. Competing-risk of incident type 2 diabetes against risk of participant mortality**

|  |  | **Percentile** | **Cases % (N)** | **HR [95% CI]** | **p-value** |
| --- | --- | --- | --- | --- | --- |
| Model 1 | Night | 0-50% (ref.) | 1∙98 (835) | ∙∙ | ∙∙ |
| N = 84,510 |  | 50-70% | 2∙52 (426) | 1∙32 [1∙18-1∙49]* | <0∙0001 |
|  |  | 70-90% | 2∙72 (459) | 1∙44 [1∙28-1∙61]* | <0∙0001 |
|  |  | 90-100% | 3∙16 (267) | 1∙66 [1∙44-1∙91]* | <0∙0001 |
|  | Day | 0-50% (ref.) | 2∙41 (1017) | ∙∙ | ∙∙ |
|  |  | 50-70% | 2∙43 (410) | 0∙97 [0∙87-1∙09] | 0∙61 |
|  |  | 70-90% | 2∙22 (375) | 0∙85 [0∙75-0∙95]* | 0∙006 |
|  |  | 90-100% | 2∙19 (185) | 0∙73 [0∙63-0∙86]* | 0∙00014 |
| Model 2 | Night | 0-50% (ref.) | 1∙97 (818) | ∙∙ | ∙∙ |
| N = 83,052 |  | 50-70% | 2∙47 (411) | 1∙28 [1∙13-1∙44]* | <0∙0001 |
|  |  | 70-90% | 2∙70 (449) | 1∙41 [1∙26-1∙58]* | <0∙0001 |
|  |  | 90-100% | 3∙14 (261) | 1∙59 [1∙38-1∙83]* | <0∙0001 |
|  | Day | 0-50% (ref.) | 2∙38 (987) | ∙∙ | ∙∙ |
|  |  | 50-70% | 2∙41 (400) | 0∙99 [0∙88-1∙11] | 0∙86 |
|  |  | 70-90% | 2∙23 (371) | 0∙88 [0∙78-1∙00]* | 0∙044 |
|  |  | 90-100% | 2∙18 (181) | 0∙78 [0∙66-0∙91]* | 0∙0021 |
| Model 3 | Night | 0-50% (ref.) | 1∙96 (786) | ∙∙ | ∙∙ |
| N = 80,181 |  | 50-70% | 2∙46 (394) | 1∙29 [1∙14-1∙45]* | <0∙0001 |
|  |  | 70-90% | 2∙69 (431) | 1∙39 [1∙23-1∙56]* | <0∙0001 |
|  |  | 90-100% | 3∙16 (253) | 1∙53 [1∙32-1∙77]* | <0∙0001 |
|  | Day | 0-50% (ref.) | 2∙35 (942) | ∙∙ | ∙∙ |
|  |  | 50-70% | 2∙39 (384) | 1∙06 [0∙94-1∙20] | 0∙32 |
|  |  | 70-90% | 2∙26 (362) | 1∙02 [0∙90-1∙15] | 0∙75 |
|  |  | 90-100% | 2∙20 (176) | 0∙98 [0∙83-1∙16] | 0∙84 |

Data are proportional sub-hazards (95% CI) of type 2 diabetes, including participant mortality as a competing risk. Model covariates: model 1: age, sex, and ethnicity; model 2: model 1 covariates plus income, material deprivation, education, and employment status; and model 3: model 2 covariates plus smoking status, alcohol consumption, healthy diet, physical activity, and urbanicity. * p<·05.


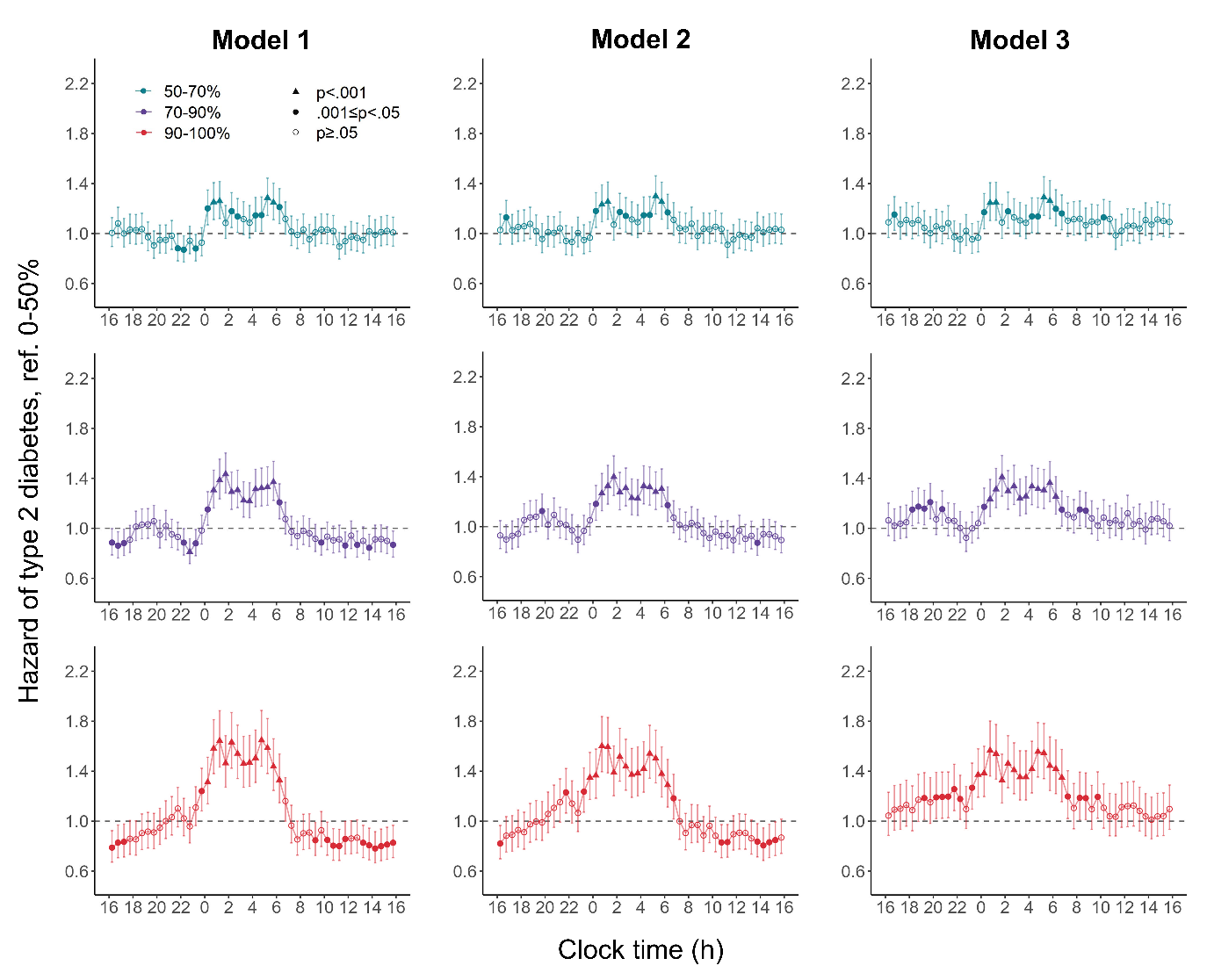
**Figure S10.** **Risk of type 2 diabetes for light exposures across twenty-four-hours.** Data are hazard ratios and vertical bars represent 95% CIs. Model covariates: model 1: age, sex, and ethnicity; model 2: model 1 covariates plus income, material deprivation, education, and employment status; and model 3: model 2 covariates plus smoking status, alcohol consumption, healthy diet, physical activity, and urbanicity. 50-70%, 70-90%, and 90-100% are percentile groups for light exposure intensity within half-hour intervals. Referent category is the 0-50th percentile group for all models.

# **Table S11. Proportional hazards assumption test for Models 1-3**

|  | **Model 1** |  | **Model 2** |  | **Model 3** |  |
| --- | --- | --- | --- | --- | --- | --- |
|  | **χ^2^ (d.f.)** | **p-value** | **χ^2^ (d.f.)** | **p-value** | **χ^2^ (d.f.)** | **p-value** |
| Night light | 3∙6 (3) | 0∙31 | 3∙1 (3) | 0∙37 | 2∙7 (3) | 0∙44 |
| Day light | 0∙88 (3) | 0∙83 | 1∙3 (3) | 0∙73 | 1∙0 (3) | 0∙79 |
| Age | 0∙0044 (1) | 0∙95 | 0∙023 (1) | 0∙88 | 0∙016 (1) | 0∙90 |
| Sex | 3.0 (1) | 0∙083 | 1∙8 (1) | 0∙17 | 1∙4 (1) | 0∙23 |
| Ethnicity | 1∙7 (1) | 0∙20 | 1∙2 (1) | 0∙27 | 0∙55 (1) | 0∙46 |
| Employment | ∙∙ | ∙∙ | 0∙0066 (1) | 0∙94 | 0∙22 (1) | 0∙64 |
| Income | ∙∙ | ∙∙ | 8∙5 (5) | 0∙13 | 11 (5) | 0∙053 |
| Deprivation | ∙∙ | ∙∙ | 0∙50 (1) | 0∙48 | 0∙66 (1) | 0∙42 |
| Education | ∙∙ | ∙∙ | 0∙93 (2) | 0∙63 | 0∙44 | 0∙80 |
| Physical activity | ∙∙ | ∙∙ | ∙∙ | ∙∙ | 3∙3 (1) | 0∙068 |
| Alcohol | ∙∙ | ∙∙ | ∙∙ | ∙∙ | 2∙3 (1) | 0∙13 |
| Smoking | ∙∙ | ∙∙ | ∙∙ | ∙∙ | 2∙6 (2) | 0∙27 |
| Urbanicity | ∙∙ | ∙∙ | ∙∙ | ∙∙ | 1∙2 (1) | 0∙26 |
| Diet | ∙∙ | ∙∙ | ∙∙ | ∙∙ | 0∙22 (1) | 0∙64 |
| Global | 9∙1 (9) | 0∙42 | 18 (18) | 0∙45 | 27 (24) | 0∙30 |

χ2 (d.f.) and p-values assessed whether the gradient of scaled Schoenfeld residuals vs. time significantly differed from zero, for each model covariate and for each model (Global). Proportional hazards assumption was tested using ‘cox.zph’ in R.

# **S12. Supplementary Methods**

**Protocol**

A cohort of approximately 502,000 participants were assessed at one of twenty-two assessment centres across the UK between 2006-2010. From this cohort of 502,000, invitations to wear an AX3 device for one week were accepted by 103,669 (out of 236,519 invitations sent via post) between 2013-2016. Recruitment and consent procedures are documented in detail on the UK Biobank website (links provided in Table S1).

**Implementation of Cox proportional hazards models**

Cox proportional hazards models were implemented using the ‘survival’ package in R (version 4·1·0). Example model syntax for implemented Cox models is as follows:

*coxph(Surv(years, diabetes_diagnosis) ~ light_night + light_day + age + sex + ethnicity, data=data)*

where *years* represented time between light recording and either diabetes diagnosis, death, or study endpoint (whichever came first), and *diabetes_diagnosis* was a binary marker indicating whether each participant was diagnosed with type 2 diabetes.

For categorical variables, contrasts were set using the default ‘contr.treatment’ setting in R, that defines a referent category for comparison. The proportional hazards assumption was assessed with the ‘cox.zph’ function in R. Cumulative incidence curves were adjusted using the ‘survminer’ package with a ‘conditional’ approach.

Proportional sub-hazards models for competing-risks were implemented using the ‘cmprsk’ package in R. An example of model syntax for proportional sub-hazards models is as follows:

*crr(ftime = years, fstatus = diabetes_diagnosis, cov1 = covariates, failcode = n)*

where *years* represents time between light recording and either death or censoring, *diabetes_diagnosis* is a numerical representation of diabetes diagnosis or participant mortality (0 = living, 1 = diabetes diagnosis, 2 = mortality), *covariates* is a data structure containing age, sex, ethnicity, and other covariates, and *n* represents the survival event (diabetes diagnosis or mortality).

**Light data processing**

Light data from Axivity AX3 devices were downsampled to 1Hz, converted using 10^(device output/341) according to device manual instructions, and averaged into 10-second mean epochs. After cleaning of light data according to non-wear, the median of all epochs within 10-minute rolling windows was extracted, stepped in 2-minute increments, to increase speed of data processing. Participant-level data cleaning was conducted using twenty-four hour profiles, to ensure participants’ data were high quality and captured light exposure across both the night and day. Participants were required to have ≥60 of 210 possible minutes of cumulative light data across the week within each half-hour clock-time range, and 6,761 of 95,665 were excluded. A final sample of 88,904 participants had night and day light values.

**Night light and day light factors**

As reported previously,^2^ night light (00:30-06:00) and day light (07:30-20:30) factors were extracted using factor analysis, with factor loading ≥0·5, varimax rotation (cumulative proportion of variance explained = 0·56). Day and night light factors exhibited internal consistency (Cronbach’s α=.98 and α=.93, respectively), and had a weak positive correlation (r_s_=0·10, p<·0001).

**Circadian rhythm modelling**

Circadian rhythms were modelled from light data using a two-stage model of the human circadian system, consisting of ‘Process L’, and ‘Process P’.

*Process L:* Light data were input to a dynamic stimulus processer representing transduction and transmission of light from the environment to the central circadian pacemaker, via photoreceptors and the retino-hypothalamic tract. Process L was modelled as follows:

$$\dot{n}=C\left( \alpha\left( 1-n \right) - \beta n \right)$$

$$\alpha= \alpha_{0}\left( \frac{I}{I_{0}} \right)^{p}$$

where $I$ represents light intensity, and $n$ represents the fraction of saturated photoreceptor elements,.

*Process P:* Output from Process L was input to a limit cycle oscillator, representing the central circadian pacemaker. Process P was modelled as follows:

$$\dot{x}= \frac{\pi}{12}\left( x_{c}+\hat{B}\left( 1-0\cdot4x \right)\left( 1-0\cdot4x_{c} \right) \right)$$

$$\dot{x}_{c}=\frac{\pi}{12}\left( \mu\left( x_{c}-\frac{{4x}_{c}^{3}}{3} \right)- x\left( \left( \frac{24}{0\cdot99729\tau_{x}} \right)^{2}+ k\hat{B}\left( 1-0\cdot4x \right)\left( 1-0\cdot4x_{c} \right) \right) \right)$$

$$\hat{B}=G\left( 1-n \right)\alpha$$

where $x$ and $x_{c}$ represent the state of the central circadian pacemaker, and $\hat{B}$ represents the drive on the pacemaker of transmission of light information from Process L.

Light intensity $(I)$, photoreceptor state $(n)$, and circadian pacemaker state $(x, x_{c})$ are all functions of time, defined at every light-recording epoch.

Fixed parameters were:

$$C=60$$

$$\beta=0\cdot013 {min}^{-1}$$

$$\alpha_{0}=0\cdot16 {min}^{-1}$$

$$I_{0}=9500$$

$$p=0\cdot6$$

$$G=19\cdot875$$

$$\mu=0\cdot23$$

$$\tau_{x}=24\cdot2$$

$$k=0\cdot55$$

Amplitude was calculated at each epoch, as follows:

$$A=\sqrt{x^{2}+x_{c}^{2}}$$

for all epoch-pairs of $x$ and $x_{c}$. Mean, minimum, and maximum amplitude were extracted across all epoch-by-epoch amplitude data for each participant.

Phase was calculated as clock time at the minimum $x$ value in each 24h interval, plus a reference value of 0·8h. Phase was calculated for each day of data, and the intra-individual mean and standard deviation of phase were calculated for each participant.

This modelling approach required consecutive epochs as input, but light data contained missing epochs due to device non-wear. We therefore imputed missing light data of intervals ≤120 min using Kalman imputation with the ‘imputeTS’ package in R. We then extracted the longest continuous interval of light data for each participant containing whole days only, and excluded all participants with <3 continuous days of light data (95,068 remaining after exclusion). Participant-level exclusions for low-quality light data were also applied, as described above. A final sample of participants had suitable light data for modelling circadian rhythms. The majority of participants in this sample had 6 days of data remaining (65·8%), and there were 5 days in 21·2%, 4 days in 6·9%, and 3 days in 6·0%. Imputation was not required in 27% of participants, and a median (IQR) of 0·57 (0·80) h of data were imputed the remaining 73%.

Initial conditions for Process P were defined as the values of $x$ and $x_{c}$ on the limit cycle, at the clock time of participant’s average midsleep. We minimized the transient effects of initial conditions on model outputs by replicating each participant’s data to 35 days in length (e.g., seven days of light data were replicated five times, concatenated, and input to the model as an interval of 35 days). Phase and amplitude were calculated using the last N days of the replicated times series, where N was number of days of data available for each participant.

**Sleep estimation**

Sleep was estimated using GGIR,^3,4^ a validated open-source R package for estimating sleep-wake state from accelerometer data, as reported previously.^5,6^ GGIR calculates sleep duration using the total duration of sustained inactivity between algorithmically defined ‘sleeponset’ and ‘wakeup’ times. Sustained inactivity is defined as <5° of deviation of the accelerometer along its z-axis for >5 min. Sleep duration was calculated as the average duration across all available nights for each participant.

**Ancestry definition and principle components of ancestry**

European ancestry classification was undertaken using the Human Genome Diversity Project-1000 Genomes (HGDP-1KG) harmonized reference dataset.^7^ The HGDP-1KG is a high quality dataset of 4,094 whole genomes from labelled diverse continental populations. Principal components analysis (PCA) was performed on unrelated (KING kinship coefficient < 0∙125) individuals after pruning variants (500kb window, r2 = 0∙01) to extract the top 10 PCs of ancestry.^8-10^ We then projected individuals from the UK Biobank onto the HGDP-1KG PC space and trained a random forest classifier given continental ancestry labels from the HGDP-1KG cohort to assign ancestry to UK Biobank individuals based on their top 10 PC scores. The minimum random forest probability for assignment to a particular ancestry group was 0∙5 and we completed 20 iterations of this model. An individual was assigned to the European ancestry group and included in genetic cox models if 20/20 iterations assigned them to the European ancestry, otherwise individuals were excluded as non-European or admixed. Finally, PCA was completed within European UKB individuals to extract the top 5 PCs of ancestry for inclusion as population stratification covariates in genetic cox models.

**References**

1. Said MA, Verweij N, van der Harst P. Associations of combined genetic and lifestyle risks with incident cardiovascular disease and diabetes in the UK Biobank Study. *JAMA cardiology* 2018; **3**(8): 693-702.

2. Burns AC, Windred DP, Rutter MK, et al. Day and night light exposure are associated with psychiatric disorders: an objective light study in> 85,000 people. *Nature Mental Health* 2023: 1-10.

3. Migueles JH, Rowlands AV, Huber F, Sabia S, van Hees VT. GGIR: a research community–driven open source R package for generating physical activity and sleep outcomes from multi-day raw accelerometer data. *Journal for the Measurement of Physical Behaviour* 2019; **2**(3): 188-96.

4. van Hees VT, Sabia S, Jones SE, et al. Estimating sleep parameters using an accelerometer without sleep diary. *Scientific reports* 2018; **8**(1): 1-11.

5. Windred DP, Jones SE, Russell A, et al. Objective assessment of sleep regularity in 60 000 UK Biobank participants using an open-source package. *Sleep* 2021; **44**(12): zsab254.

6. Windred DP, Burns AC, Lane JM, et al. Sleep regularity is a stronger predictor of mortality risk than sleep duration: A prospective cohort study. *Sleep* 2023: zsad253.

7. Koenig Z, Yohannes MT, Nkambule LL, et al. A harmonized public resource of deeply sequenced diverse human genomes. *bioRxiv* 2023.

8. Patterson N, Price AL, Reich D. Population structure and eigenanalysis. *PLoS genetics* 2006; **2**(12): e190.

9. Purcell S, Neale B, Todd-Brown K, et al. PLINK: a tool set for whole-genome association and population-based linkage analyses. *The American journal of human genetics* 2007; **81**(3): 559-75.

10. Manichaikul A, Mychaleckyj JC, Rich SS, Daly K, Sale M, Chen W-M. Robust relationship inference in genome-wide association studies. *Bioinformatics* 2010; **26**(22): 2867-73.

# **S13. STROBE Statement**

**STROBE Statement—Checklist of items that should be included in reports of *cohort studies***

|  | Item No | Recommendation | Manuscript Section |
| --- | --- | --- | --- |
| **Title and abstract** | 1 | (*a*) Indicate the study’s design with a commonly used term in the title or the abstract | - Abstract |
|  |  | (*b*) Provide in the abstract an informative and balanced summary of what was done and what was found | - Abstract |
| Introduction | | |  |
| Background/rationale | 2 | Explain the scientific background and rationale for the investigation being reported | - Abstract - Introduction paragraphs 1-2 |
| Objectives | 3 | State specific objectives, including any prespecified hypotheses | - Abstract - Introduction paragraph 3 |
| Methods | | |  |
| Study design | 4 | Present key elements of study design early in the paper | - Abstract - Introduction paragraph 3 - Methods: Overview |
| Setting | 5 | Describe the setting, locations, and relevant dates, including periods of recruitment, exposure, follow-up, and data collection | - Abstract - Methods: Overview - Results: Descriptive statistics |
| Participants | 6 | (*a*) Give the eligibility criteria, and the sources and methods of selection of participants. Describe methods of follow-up | - Methods: Overview |
|  |  | (*b*) For matched studies, give matching criteria and number of exposed and unexposed | - N/A |
| Variables | 7 | Clearly define all outcomes, exposures, predictors, potential confounders, and effect modifiers. Give diagnostic criteria, if applicable | - Methods: Overview - Methods: ‘Exposure: Light and modeled circadian rhythms’ - Methods: ‘Outcome: Incident type 2 diabetes’ - Methods: Covariates - Supplementary S2-3 |
| Data sources/ measurement | 8* | For each variable of interest, give sources of data and details of methods of assessment (measurement). Describe comparability of assessment methods if there is more than one group | - Methods: Overview - Methods: ‘Exposure: Light and modeled circadian rhythms’ - Methods: ‘Outcome: Incident type 2 diabetes’ - Methods: Covariates - Supplementary S2-3 |
| Bias | 9 | Describe any efforts to address potential sources of bias | - Methods: Statistical analysis - Supplementary S5 |
| Study size | 10 | Explain how the study size was arrived at | - Methods: Overview |
| Quantitative variables | 11 | Explain how quantitative variables were handled in the analyses. If applicable, describe which groupings were chosen and why | - Methods: Statistical analysis - Supplementary S5 |
| Statistical methods | 12 | (*a*) Describe all statistical methods, including those used to control for confounding | - Methods: Statistical analysis - Supplementary S5 |
|  |  | (*b*) Describe any methods used to examine subgroups and interactions | - Methods: Statistical analysis - Supplementary S5 |
|  |  | (*c*) Explain how missing data were addressed | - Methods: ‘Exposure: Light and modeled circadian rhythms’ - Supplementary S5 |
|  |  | (*d*) If applicable, explain how loss to follow-up was addressed | - N/A |
|  |  | (*e*) Describe any sensitivity analyses | - Methods: Statistical analysis - Supplementary S4 |
| Results | | |  |
| Participants | 13* | (a) Report numbers of individuals at each stage of study—e.g., numbers potentially eligible, examined for eligibility, confirmed eligible, included in the study, completing follow-up, and analysed | - Methods: Overview - Methods: ‘Exposure: Light and modeled circadian rhythms’ - Results: Descriptive statistics - Supplementary S5 |
|  |  | (b) Give reasons for non-participation at each stage | - Methods: Overview |
|  |  | (c) Consider use of a flow diagram | - N/A |
| Descriptive data | 14* | (a) Give characteristics of study participants (e.g., demographic, clinical, social) and information on exposures and potential confounders | - Results: Descriptive statistics - Results: Table 1 |
|  |  | (b) Indicate number of participants with missing data for each variable of interest | - Methods: ‘Exposure: Light and modeled circadian rhythms’ - Results: Descriptive statistics - Supplementary S5 |
|  |  | (c) Summarise follow-up time (e.g.,, average and total amount) | - Abstract - Results: Descriptive statistics |
| Outcome data | 15* | Report numbers of outcome events or summary measures over time | - Results: Figure 1 |
| Main results | 16 | (*a*) Give unadjusted estimates and, if applicable, confounder-adjusted estimates and their precision (e.g., 95% confidence interval). Make clear which confounders were adjusted for and why they were included | - Results: Tables 2-4 - Supplementary S4 - Methods: Statistical analysis |
|  |  | (*b*) Report category boundaries when continuous variables were categorized | - Results: Figure 1 |
|  |  | (*c*) If relevant, consider translating estimates of relative risk into absolute risk for a meaningful time period | - N/A |
| Other analyses | 17 | Report other analyses done—e.g., analyses of subgroups and interactions, and sensitivity analyses | - Supplementary S4 |
| Discussion | | |  |
| Key results | 18 | Summarise key results with reference to study objectives | - Discussion paragraph 1 |
| Limitations | 19 | Discuss limitations of the study, taking into account sources of potential bias or imprecision. Discuss both direction and magnitude of any potential bias | - Discussion paragraph 6 |
| Interpretation | 20 | Give a cautious overall interpretation of results considering objectives, limitations, multiplicity of analyses, results from similar studies, and other relevant evidence | - Discussion paragraphs 2-5, 7 |
| Generalisability | 21 | Discuss the generalisability (external validity) of the study results | - Discussion paragraph 6 |
| Other information | | |  |
| Funding | 22 | Give the source of funding and the role of the funders for the present study and, if applicable, for the original study on which the present article is based | - Methods: Roles of the funding source |
